# ROS scavenging nanoengineered bioactive glass interfaces reprogram macrophage immunity for tendon–bone regeneration

**DOI:** 10.1093/rb/rbag121

**Published:** 2026-06-09

**Authors:** Bowen Cai, Fanrui Zeng, Kaixiao Xue, Zhi Shen, Chang Qiao, Han Wu, Qunyi Wang, Jue Zhang, Shahin Homaeigohar, Kai Zheng, Bin Zhu, Jiahu Fang

**Affiliations:** Department of Orthopedics, the First Affiliated Hospital with Nanjing Medical University, Nanjing 210029, China; Jiangsu Institute of Functional Reconstruction and Rehabilitation, the First Affiliated Hospital with Nanjing Medical University, Nanjing 210029, China; Jiangsu Province Engineering Research Center of Stomatological Translational Medicine & Jiangsu Key Laboratory of Oral Diseases, Nanjing Medical University, Nanjing 210029, China; Affiliated Hospital of Stomatology, Nanjing Medical University, Nanjing 210029, China; Department of Orthopedics, the First Affiliated Hospital with Nanjing Medical University, Nanjing 210029, China; Jiangsu Institute of Functional Reconstruction and Rehabilitation, the First Affiliated Hospital with Nanjing Medical University, Nanjing 210029, China; Department of Orthopedics, the First Affiliated Hospital with Nanjing Medical University, Nanjing 210029, China; Jiangsu Institute of Functional Reconstruction and Rehabilitation, the First Affiliated Hospital with Nanjing Medical University, Nanjing 210029, China; Department of Orthopedics, the First Affiliated Hospital with Nanjing Medical University, Nanjing 210029, China; Jiangsu Institute of Functional Reconstruction and Rehabilitation, the First Affiliated Hospital with Nanjing Medical University, Nanjing 210029, China; Jiangsu Province Engineering Research Center of Stomatological Translational Medicine & Jiangsu Key Laboratory of Oral Diseases, Nanjing Medical University, Nanjing 210029, China; Affiliated Hospital of Stomatology, Nanjing Medical University, Nanjing 210029, China; Nanjing Stomatological Hospital, Affiliated Hospital of Medical School, Research Institute of Stomatology, Nanjing University, Nanjing 210029, China; Anhui Province Engineering Research Center for Dental Materials and Application, School of Stomatology, Wannan Medical College, Wuhu 241002, China; School of Science and Engineering, University of Dundee, Dundee DD1 4HN, UK; Jiangsu Province Engineering Research Center of Stomatological Translational Medicine & Jiangsu Key Laboratory of Oral Diseases, Nanjing Medical University, Nanjing 210029, China; Affiliated Hospital of Stomatology, Nanjing Medical University, Nanjing 210029, China; Department of Orthopedics, the First Affiliated Hospital with Nanjing Medical University, Nanjing 210029, China; Jiangsu Institute of Functional Reconstruction and Rehabilitation, the First Affiliated Hospital with Nanjing Medical University, Nanjing 210029, China; Department of Orthopedics, the First Affiliated Hospital with Nanjing Medical University, Nanjing 210029, China; Jiangsu Institute of Functional Reconstruction and Rehabilitation, the First Affiliated Hospital with Nanjing Medical University, Nanjing 210029, China

**Keywords:** tendon–bone interface, immunomodulation, cerium-doped bioactive glass nanoparticles, macrophage polarization, surface modification, rotator cuff repair

## Abstract

Failure of rotator cuff repair largely stems from ineffective regeneration of the tendon–bone interface (TBI), driven by persistent inflammation and excessive reactive oxygen species (ROS) accumulation at the biomaterial tissue interface. Here, we engineer an immunoregulatory suture interface by incorporating cerium-doped mesoporous bioactive glass nanoparticles (Ce-MBGNs) into a gelatin/tannic acid (Gel/TA) coating on polyethylene terephthalate sutures. The hierarchical Gel/TA/Ce-MBGN coating displays uniform morphology, robust adhesion and superior ROS scavenging activity. *In vitro*, Ce-MBGN-functionalized surfaces markedly promote osteogenic and chondrogenic differentiation of bone marrow mesenchymal stem cells, while polarizing macrophages toward an anti-inflammatory M2 phenotype and substantially reducing intracellular ROS. In a rat rotator cuff repair model, high Ce-MBGN-loaded sutures significantly enhance fibrocartilaginous enthesis regeneration, increase bone mineral density and elevate M2 macrophage infiltration at the TBI. These regenerative effects correlate with suppressed HIF-1α expression *in vivo*, suggesting that localized redox modulation drives macrophage immunometabolic reprogramming to foster a pro-regenerative microenvironment. This Ce-MBGN-enabled interface engineering strategy simultaneously scavenges ROS, modulates immunity, and supports multi-lineage tissue regeneration at the TBI, offering a promising, clinically translatable approach for immunomodulatory suture design to improve rotator cuff repair outcomes.

## Graphical Abstract

**Graphical Abstract:**
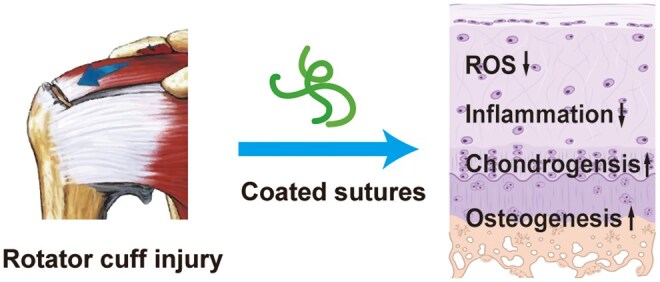

## Introduction

Rotator cuff tears (RCTs) are a prevalent musculoskeletal injury, particularly in individuals over 65 years of age, with retear rates following surgical repair ranging from 20% to 94% [1, 2]. The primary cause of surgical failure is the inability to restore the native tendon–bone interface (TBI), a complex gradient structure that connects tendon, fibrocartilage and bone [3]. Current repair techniques seldom recreate this native fibrocartilaginous enthesis, and healing often results in the formation of mechanically weak scar tissue [4]. Recent studies indicate that this failure results from both structural defects and an abnormal inflammatory microenvironment. Specifically, excessive accumulation of reactive oxygen species (ROS) at the injury site disrupts immune homeostasis and delays the resolution of inflammation [5, 6].

The plasticity of macrophages plays a crucial role in TBI healing. A timely transition from the pro-inflammatory M1 phenotype to the pro-regenerative M2 phenotype is essential for effective tissue remodeling [7]. However, within the challenging microenvironment of RCTs, macrophages are often trapped in a state of metabolic dysfunction. Elevated levels of ROS have been shown to aberrantly stabilize hypoxia-inducible factor-1α (HIF-1α), even under normoxic conditions [8]. The sustained activation of the ROS/HIF-1α axis forces macrophages to maintain a glycolysis-dominant metabolic profile, thereby locking them in the M1 phenotype and preventing their repolarization toward M2 [9, 10]. Consequently, this immunometabolic stagnation perpetuates chronic inflammation and inhibits fibrocartilage regeneration [11]. Thus, therapeutic strategies that provide only structural support are inadequate; there is an urgent need for bioactive interventions capable of actively scavenging ROS to disrupt this HIF-1α-mediated metabolic dysfunction and restart the regenerative process. PET is commonly used in synthetic materials for rotator cuff repair due to its high tensile strength and hydrolytic stability [12, 13]. However, its bio-inert and hydrophobic nature limits its integration with native tissue [14]. To address this, surface engineering modifications, including silk fibroin, hyaluronic acid and hydroxyapatite, have been employed to improve biocompatibility and osteoconduction [15–17]. While these modifications enhance cell–material interactions, persistent oxidative stress and metabolic dysregulation at the injury site continue to present significant challenges. This underscores the potential to complement these structural improvements with active immunomodulatory strategies, particularly those targeting the ROS/HIF-1α axis, to optimize the regenerative environment.

Functionalizing suture surfaces with bioactive nanomaterials presents a promising strategy to modulate the local microenvironment. In this study, cerium-doped mesoporous bioactive glass nanoparticles (Ce-MBGNs) were selected for their dual functionality [18, 19]. Cerium exhibits a reversible Ce^3+^/Ce^4+^ redox couple, providing antioxidant capacity that mitigates ROS accumulation associated with injury [20]. By combining these properties, this study aimed to evaluate whether such a composite coating could regulate macrophage polarization via the ROS/HIF-1α axis while simultaneously supporting multi-tissue regeneration required for TBI healing.

**Figure 1:**
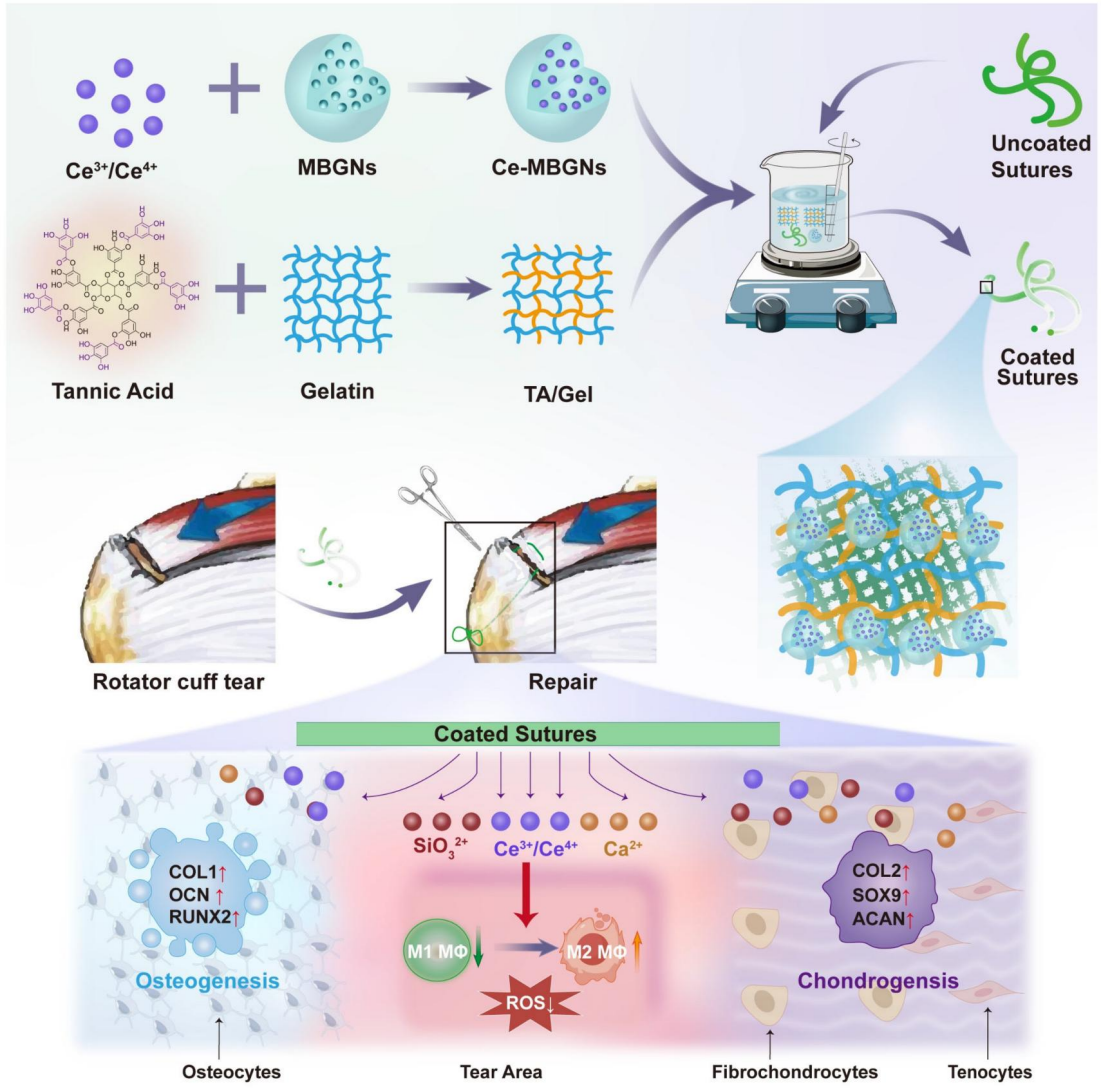
Schematic illustration of the preparation process of the sutures coated with Ce-MBGN-incorporated Gel/TA hydrogel for TBI regeneration. This approach promotes cartilage and osteogenic differentiation of BMSCs, accelerates polarization of anti-inflammatory M2 macrophages, and clears ROS, thereby facilitating the simultaneous regeneration of soft and hard tissues at TBI.

In this study, we present a novel immunometabolic reprogramming strategy for TBI repair by engineering a functionalized PET suture interface. A composite coating composed of gelatin (Gel) and tannic acid (TA) was designed to securely anchor Ce-MBGNs onto the inert PET surface. The Gel/TA matrix enhances the hydrophilicity of the suture surface and provides additional antioxidant capacity via the phenolic groups of TA. We hypothesized that this nanoengineered interface would reprogram macrophage immunometabolism by scavenging ROS and modulating the HIF-1α pathway, thereby fostering a pro-regenerative microenvironment conducive to multi-tissue regeneration. In this context, the translational potential of this approach lies primarily in its utility as a surface-functionalization strategy for existing, clinically approved PET sutures. By leveraging pre-existing materials with established mechanical stability, this interface-engineering method offers a straightforward path toward clinical application without the need to redesign the bulk properties of the suture. We systematically evaluate the potential of these functionalized sutures to drive macrophage polarization and promote the dual osteogenic and chondrogenic differentiation of bone marrow mesenchymal stem cells (BMSCs) *in vitro*. Furthermore, their therapeutic efficacy is validated *in vivo* by assessing fibrocartilaginous enthesis regeneration in a rat RCT model (Figure 1).

## Materials and methods

### Synthesis of Ce-MBGNs

Ce-MBGNs were prepared using a microemulsion-assisted sol-gel process followed by post-impregnation. Briefly, 2.8 g of cetrimonium bromide (CTAB, Millipore, 99%, China) was dissolved in 132 mL of deionized water under continuous stirring at 35°C. Once CTAB was fully dissolved, as indicated by the formation of a clear solution, 40 mL of ethyl acetate (Millipore, 99.7%, China) was added, and the temperature was adjusted to 25°C. After stirring for 30 min, 28 mL of aqueous ammonia (1 M, Aladdin, China), prepared by adding 3.66 mL of ammonia solution (28%) and diluting to 28 mL with water, was introduced. The mixture was stirred for an additional 15 min. Next, 14.4 mL of tetraethyl orthosilicate (Millipore, 99%, China) was added. After 30 min, 9.12 g of calcium nitrate tetrahydrate (Ca(NO_3_)_2_·4H_2_O, Aladdin, China) was included, and the resulting solution was stirred for 4 h. The formed whitish precipitate was collected through centrifugation, washed twice with deionized water and once with 75% ethanol. The collected precipitate was then dried at 60°C overnight. Subsequently, 800 mg of dried particles were soaked in 50 mL of a cerium nitrate ethanol solution (Ce(NO_3_)_3_, Millipore, 99.5%, China) (0.01 M) under stirring for 4 h at room temperature. After impregnation, the treated particles were washed twice with ethanol, with centrifugation for 15 min each time, before being dried at 60°C overnight. The dried particles were calcined in air at 700°C for 3 h, with a heating rate of 2°C/min, in a furnace to obtain Ce-MBGNs. All chemicals were used as received without further purification.

### Coating of PET sutures

PET sutures (Ethicon, USA) were pre-treated to enhance surface reactivity before coating. Sutures were ultrasonically cleaned in 75% ethanol for 2 h (frequency: 40 kHz), followed by drying at 60°C overnight in a vacuum oven. Surface hydrolysis was induced by immersing the cleaned sutures in 2.5 M NaOH (aq) with stirring for 3 h at room temperature, followed by rinsing with deionized water (three cycles) to remove residual alkali. Previous studies have demonstrated that this NaOH pretreatment enhances surface reactivity while maintaining the mechanical strength of PET fibers, ensuring that the fibers remain mechanically stable [21, 22].

Four experimental groups of coated sutures were prepared: (i) blank control (untreated PET sutures), (ii) Gel/TA coating, (iii) Gel/TA + 10%BG (low-dose Ce-MBGNs) and (iv) Gel/TA + 30%BG (high-dose Ce-MBGNs). Coating solutions were prepared as follows: Ce-MBGNs (0, 50 or 150 mg) were dispersed in 3 mL of deionized water by ultrasonication (40 kHz, 15 min) to yield suspensions at 0, 16.7 or 50 mg/mL, respectively. These suspensions were then mixed with 7 mL of a 3 wt% Gel/TA solution, composed of 1 mL of TA (15 mg/mL; Sigma-Aldrich) and 6 mL of gelatin (70 mg/mL; Type B, Sigma-Aldrich), and stirred at room temperature for 3 h. Pre-treated sutures were immersed in these mixtures, stirred for 3 h and air-dried at room temperature, yielding the respective coated sutures: Gel/TA, Gel/TA/L-Ce and Gel/TA/H-Ce.

### Characterization of Ce-MBGNs and coated sutures

The morphology and nanostructure of Ce-MBGNs and coated sutures were characterized using scanning electron microscopy (SEM, JEOL JSM-6700F) and transmission electron microscopy (TEM, FEI Tecnai G2 F20) at accelerating voltages of 5 and 200 kV, respectively. Elemental composition was determined via energy-dispersive X-ray spectroscopy (EDS) coupled with SEM (SDT 600, TA Instruments, USA). Chemical bonding on suture surfaces was analyzed using Fourier transform infrared spectroscopy (FTIR; PerkinElmer, USA) in attenuated total reflectance mode, with a resolution of 0.5 cm^−1^ over the wavenumber range 4000–600 cm^−1^. Thermal stability was assessed by thermogravimetric analysis (TGA, TA Instruments, USA) in air from 25 to 800°C (heating rate: 10°C/min). To evaluate bioactivity, coated sutures were immersed in simulated body fluid (SBF, pH 7.4) at 37°C for 7 and 14 days, and surface morphology changes were examined by SEM.

### Culture and identification of rat BMSCs

Rat BMSCs were obtained from Pricella (Wuhan, China) and cultured in 25 cm^2^ flasks (Corning, USA) with alpha-modified Eagle’s medium (α-MEM; Invitrogen, USA) supplemented with 10% fetal bovine serum (FBS; Gibco, USA) and 1% penicillin–streptomycin (Gibco, USA). Cells were maintained at 37°C in a humidified incubator with 5% CO_2_. After expansion to passage 3 (P3), BMSCs were used for all *in vitro* experiments.

To confirm BMSC identity, surface markers (CD44, CD90, CD45 and CD34) were analyzed by flow cytometry. For multilineage differentiation, BMSCs were seeded at 1 × 10^5^ cells per well in six-well plates and grown to 70–80% confluence. Osteogenic differentiation was induced with 2 mL of osteogenic medium (Cyagen, China), chondrogenic differentiation with 2 mL of chondrogenic medium, and adipogenic differentiation with 2 mL of adipogenic medium. The media were refreshed every 3 days, and differentiation was maintained for 14 days at 37°C with 5% CO_2_. Differentiated cells were fixed in 4% formalin and stained with Alizarin Red S (ARS) for osteogenesis, Alcian Blue for chondrogenesis and Oil Red O for adipogenesis, followed by microscopic evaluation.

### Cell viability assay

To assess suture cytotoxicity, 2 cm suture samples (control, Gel/TA, Gel/TA/L-Ce and Gel/TA/H-Ce) were immersed in 1 mL of complete α-MEM for 14 days. Extract media were sterilized using 0.22-μm polytetrafluoroethylene filters. BMSCs were seeded at 1.25 × 10^4^ cells per well in 96-well plates and cultured overnight in complete α-MEM. The medium was then replaced with suture extracts. Cell viability was measured on days 1, 3 and 5 using a Cell Counting Kit-8 (CCK-8; Solarbio, China) assay. After adding 20 μL of CCK-8 solution and incubating for 1 h at 37°C, absorbance was recorded at 450 nm with a microplate reader (Multiskan FC, Thermo Scientific, USA). For live/dead staining, BMSCs (5 × 10^4^ cells) were seeded in 24-well plates with extract media and stained with an AM/PI kit (Bio-Tek, USA) on days 1 and 5. Live (green) and dead (red) cells were imaged and quantified using a Bio-Tek Cytation system. For adhesion studies, 5 cm sterilized sutures were placed in 24-well plates, seeded with 5 × 10^4^ BMSCs and cultured for 3 days. Cells were fixed, stained with phalloidin (cytoskeleton) and 4',6-diamidino-2-phenylindole (DAPI) (nuclei) and imaged via confocal laser scanning microscopy.

### Cell migration and invasion assay

BMSC migration was evaluated using scratch and transwell assays. For the scratch assay, BMSCs were seeded at 1 × 10^5^ cells per well in six-well plates and grown to confluence in complete α-MEM. Three parallel scratches were made with a 200 μL pipette tip, and wells were washed thrice with phosphate buffered saline (PBS). Extract media were added, and cell migration was imaged at 0, 6, 12 and 24 h using an inverted microscope (Olympus CKX53, Japan). Migration rate was calculated as follows:


(1) Migration rate percentage (%)=Wi-WtWi×100%

For the invasion assay, transwell chambers were coated with Matrigel. BMSCs (2.5 × 10^4^ cells) were seeded in the upper chamber with serum-free medium, while the lower chamber contained serum-free extract medium. After 24 h, non-invading cells were removed, and invading cells were fixed with 5% glutaraldehyde, stained with crystal violet and counted under an inverted microscope.

### Osteogenic differentiation assay

Sutures (control, Gel/TA, Gel/TA/L-Ce and Gel/TA/H-Ce) were soaked in osteogenic induction medium (OriCell, China) at 2 cm/mL for 14 days. Extracts were sterilized with 0.22 μm Teflon filters. BMSCs (5 × 10^4^ cells) were seeded in 24-well plates, and osteogenic extract media were replaced every 2 days. Alkaline phosphatase (ALP) staining and activity were assessed at 7 and 14 days, respectively, using a BCIP/NBT kit and an ALP assay kit (Beyotime, China), with PBS washes after staining. ARS staining was performed at 14 and 21 days; calcium deposits were dissolved in hexadecylpyridinium chloride, and absorbance was measured at 565 nm. Osteogenic marker expression was quantified after 7 days via reverse transcription quantitative PCR (RT-qPCR) and immunofluorescence, with imaging on an inverted fluorescence microscope.

### Chondrogenic differentiation assay

Chondrogenic extract media were prepared as described for osteogenic extracts. BMSCs (5 × 10^5^ cells) were pelleted by centrifugation at 500 × g for 5 min, resuspended in chondrogenic extract medium and cultured for 21 days with media changes every 3 days. Pellets were fixed in 4% paraformaldehyde, paraffin-embedded, sectioned and stained with Alcian Blue. Particle volume and Bern scores were calculated to assess chondrogenesis. Separately, BMSCs (5 × 10^4^ cells) were seeded in 24-well plates with chondrogenic extract medium. After 7 days, chondrogenic marker expression was evaluated by RT-qPCR and immunofluorescence, with imaging as above.

### Macrophage polarization

To assess the immunomodulatory effects of suture coatings, RAW264.7 murine macrophages (Pricella, China) were cultured in extract medium derived from various suture groups. Sutures were immersed in Dulbecco’s Modified Eagle Medium at a ratio of 2 cm/mL for 2 weeks to prepare the extract. RAW264.7 cells were seeded in 12-well plates and exposed to the extract medium supplemented with either 100 ng/mL lipopolysaccharide (LPS) to induce M1 polarization or 40 ng/mL interleukin-4 (IL-4) and 40 ng/mL interleukin-13 (IL-13) to induce M2 polarization. After 24 h, cells were harvested, blocked with 5% bovine serum albumin for 30 min and stained with fluorescein isothiocyanate (FITC)-conjugated F4/80 (123107, Biolegend, USA), phycoerythrin-cyanine 7 (PE/Cy7)-conjugated CD11b (101215, Biolegend, USA) and allophycocyanin (APC)-conjugated CD86 (105011, Biolegend, USA) antibodies. Following fixation and permeabilization with intracellular staining buffer, cells were incubated with PE-conjugated CD206 (141705, Biolegend, USA) antibody. Flow cytometry was performed to quantify macrophage populations, with F4/80+CD11b+ cells gated to assess expression of CD86 (M1 marker) and CD206 (M2 marker).

For immunofluorescence, RAW264.7 cells were seeded in 24-well plates under identical conditions, fixed with 4% paraformaldehyde for 30 min, permeabilized with 0.5% Triton X-100 (Solarbio, China) for 30 min and blocked with a commercial solution (Beyotime, China) for 1 h. Cells were incubated overnight at 4°C with primary antibodies (Abcam, USA) targeting polarization markers, followed by a 2-h incubation at 37°C with secondary antibodies (Abcam, USA). Nuclei were stained with DAPI (Beyotime, China) for 5 min, and images were acquired using an inverted fluorescence microscope (model unspecified).

### Antioxidant activity assessment

ROS levels in M1-polarized macrophages were measured using the 2',7'-dichlorodihydrofluorescein diacetate (DCFH-DA) assay (MCE, China). RAW264.7 cells were cultured in suture-derived extract medium with 100 ng/mL LPS in 24-well plates for 24 h. Cells were then incubated with 5 μM DCFH-DA in serum-free medium for 30 min at 37°C in the dark. Fluorescence images were captured under consistent conditions using an inverted fluorescence microscope. Rosup-treated cells served as a positive control and untreated cells as a negative control. To evaluate intracellular ROS scavenging, RAW264.7 cells were cultured in 6-well plates for 24 h, harvested and analyzed via RT-qPCR for expression of inflammation-related genes.

### RT-qPCR

Total RNA was extracted from BMSCs and RAW264.7 cells using an RNA purification kit (Thermo Fisher, USA) and reverse-transcribed into cDNA (1 μg per sample) with a reverse transcription kit (AG11705, Accurate Biology, China). Quantitative PCR was performed using 2× SYBR Green qPCR Master Mix on a Real-Time PCR System (AG11701, Accurate Biology, China), with a thermal profile of 95°C for 2 min, followed by 40 cycles of 95°C for 15 s and 60°C for 30 s. Glyceraldehyde-3-phosphate dehydrogenase was used as the reference gene. Target genes included COL1A1, OCN, RUNX2, COL2A1, ACAN, SOX9, TNF-α, IL-1β and CCL-2, with primers sourced from Tsingke Biotechnology (China). Relative gene expression was calculated using the ΔΔCT method. Primer sequences are provided in Supplementary Table S1.

### Animal model and surgical procedure

All animal experiments were approved by the Institutional Animal Care and Use Committee of Nanjing Medical University (IACUC-2302048). Forty-eight male Sprague-Dawley rats (8 weeks old, 250 ± 50 g) were randomly assigned to four groups (*n* = 12 per group): (i) PET suture repair (control), (ii) Gel/TA-coated PET suture, (iii) Gel/TA/L-Ce-coated PET suture and (iv) Gel/TA/H-Ce-coated PET suture. Rats were anesthetized with pentobarbital, and the right supraspinatus tendon was exposed via a longitudinal anterolateral shoulder incision. The tendon was detached from the greater tubercle of the humerus, and a double-bone tunnel was drilled. The tendon was reattached using the designated sutures passed through the tunnel, and the incision was closed. Rats were euthanized at 4 weeks (*n* = 6 per group) or 8 weeks (*n* = 6 per group) for subsequent analyses.

### Micro-CT analysis

SD rats were euthanized after 4 and 8 weeks post-surgery. Supraspinatus tendon–bone complexes were excised and scanned using micro-CT (270 μA, 90 kV, 18-μm voxel size). Coronal reconstructions were generated with Mimics software, and bone regeneration was quantified by bone mineral density (BMD) and bone volume fraction (BV/TV).

### Histological and immunohistochemical analysis

After micro-CT scanning, the supraspinatus tendon–humerus complex was fixed in 4% paraformaldehyde and decalcified in 0.5 M EDTA at 37°C for 4 weeks. Following dehydration, the samples were embedded in paraffin, and the sections were cut to a thickness of 4 μm. Hematoxylin and eosin (H&E), Safranin O/Fast Green and Masson’s trichrome staining were performed, and the images were captured using an optical microscope (DM4000B, Leica, Germany). The regenerated TBI was quantitatively assessed using a histological scoring system and a tendon maturation score. A higher histological score indicates better tendon-bone healing. Two independent observers conducted the histological evaluations, and the group allocations were blinded. Immunohistochemical stained tissue sections were imaged using an optical microscope, and the positive staining of CD206 and HIF-1α was analyzed semi-quantitatively using ImageJ (NIH, USA).

### Statistical analysis

Statistical significance was analyzed using one-way ANOVA with Tukey’s *post hoc* test or Student’s *t*-test in GraphPad Prism 9 (GraphPad Software, USA). Significance was set at *P < 0.05, **P < 0.01, ***P < 0.001.

## Results and discussion

### Characterization of Ce-MBGNs and sutures

**Figure 2:**
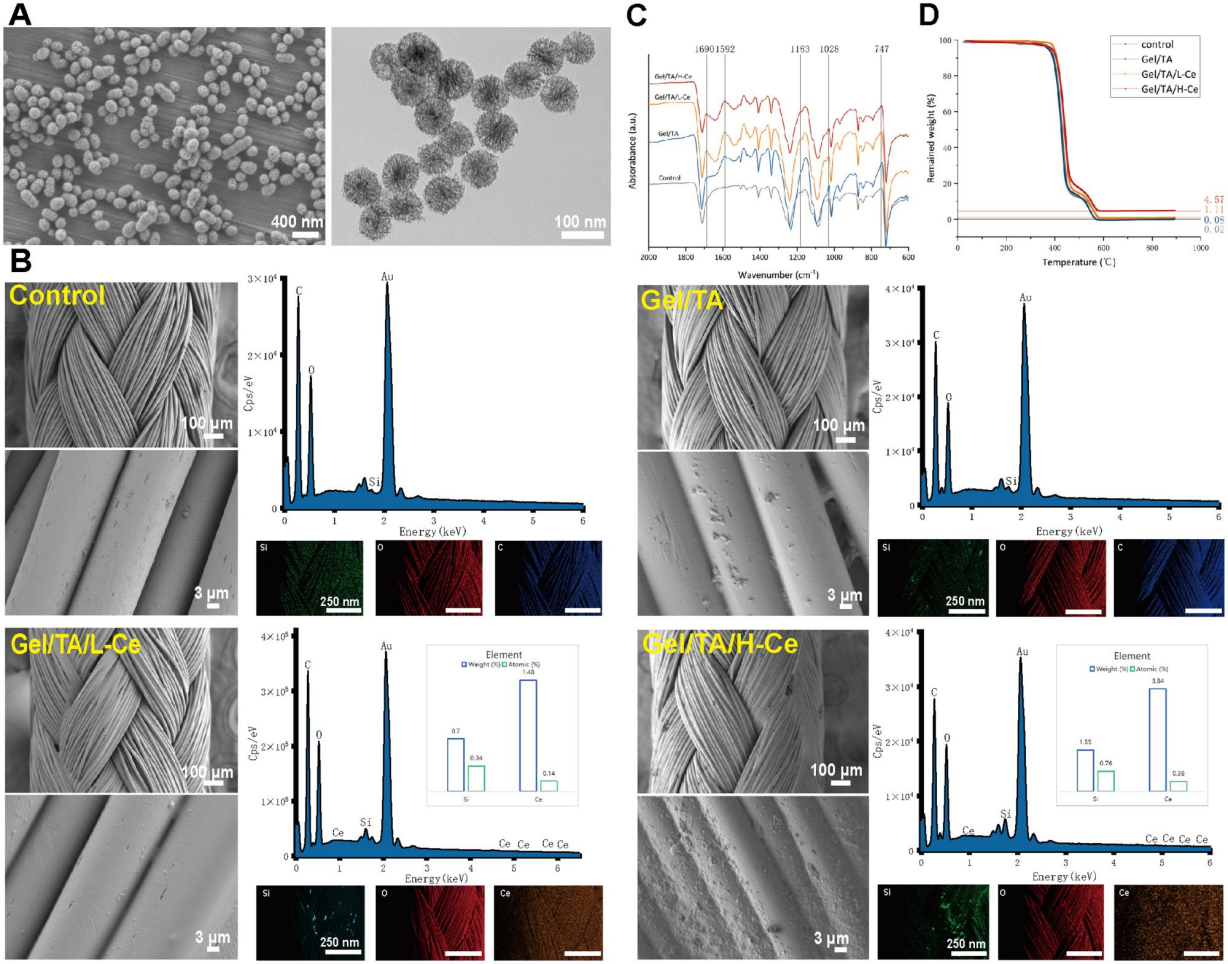
Characterization of Ce-MBGNs and sutures. (**A**) SEM and TEM images of Ce-MBGNs. (**B**) SEM images and EDS of different sutures (control, Gel/TA, Gel/TA/L-Ce and Gel/TA/H-Ce). (**C**) FTIR of different sutures. (**D**) TGA results of different sutures.

The successful synthesis of the functional payload, Ce-MBGNs, was initially confirmed through electron microscopy. SEM and TEM images revealed a monodisperse spherical morphology, a uniform particle-size distribution and a well-defined mesoporous architecture (Figure 2A). This unique mesoporous structure provides a high specific surface area, which is crucial for enhancing the interaction between the bioactive material and the surrounding biological environment.

To impart bioactivity to the inert PET sutures, we employed a Gel/TA composite matrix as an anchoring agent. SEM characterization confirmed the effectiveness of this coating strategy (Figure 2B). In contrast to the smooth surface of uncoated PET fibers, the coated groups (Gel/TA, Gel/TA/L-Ce and Gel/TA/H-Ce) exhibited a uniform layer that adhered uniformly to the fibers and bridged the interfiber gaps. This demonstrates that the Gel/TA composite matrix effectively formed a stable, adhesive interface on the chemically inert PET substrate. In the Ce-MBGNs-functionalized groups, nanoparticles were successfully anchored within this matrix, with particle density on the fiber surface increasing in proportion to the initial doping concentration.

EDS further confirmed the successful integration of the inorganic phase, demonstrating a dose-dependent increase in Ce and Si contents. The Au signal detected in the EDS spectra originated from the sputter coating applied prior to SEM observation and was not considered part of the coating composition. To elucidate the interaction mechanism between the coating components, FTIR analysis was performed (Figure 2C). The spectra exhibited PET-related absorptions, including the C = O vibration around 1690 cm^−1^ and aromatic C–H vibration around 747 cm^−1^ [23, 24]. The band around 1592 cm^−1^ was associated with aromatic C = C vibration from TA, while the absorption around 1183 cm^−1^ was related to C–O/C–N vibrations of the Gel/TA matrix [25, 26]. In the Ce-MBGN-containing groups, the absorption around 1028 cm^−1^ was assigned to Si–O–Si vibration of the bioactive glass network [27]. These results collectively support the successful introduction of the Gel/TA/Ce-MBGN coating onto PET sutures. No obvious new characteristic bands were observed, suggesting that the coating was mainly stabilized through non-covalent interactions. TGA (Figure 2D) revealed thermal stability differences of the coated sutures. The control and Gel/TA sutures degraded almost completely by 400°C, whereas Gel/TA/L-Ce and Gel/TA/H-Ce sutures retained residual mass (1.11 and 4.57%, respectively), reflecting the residual inorganic Ce-MBGN content.

**Figure 3:**
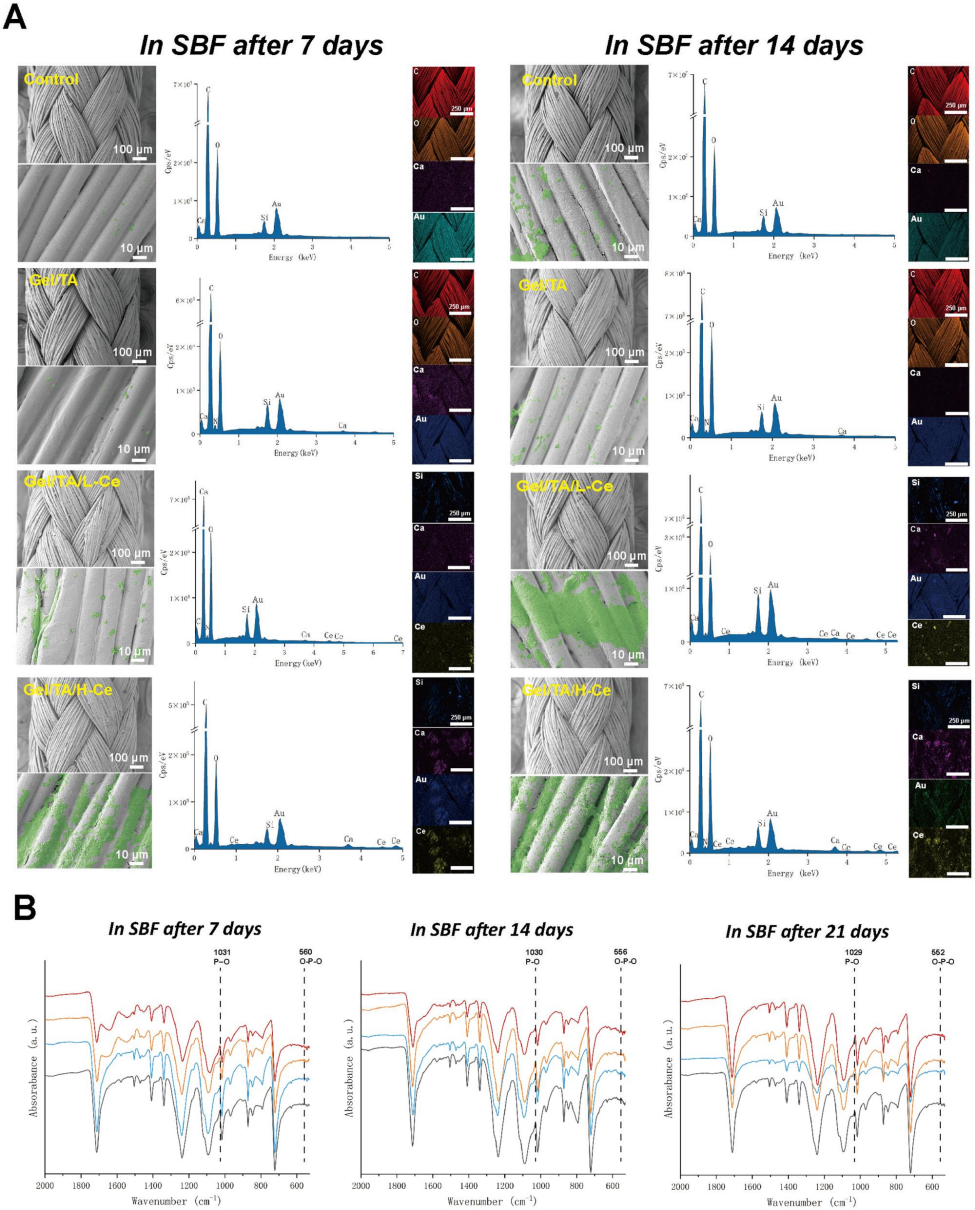
Characterization of sutures after immersion in SBF. (**A**) SEM images and EDS of different sutures (control, Gel/TA, Gel/TA/L-Ce and Gel/TA/H-Ce). (**B**) FTIR of different sutures after 7, 14 and 21 days of immersion in SBF.

Bioactivity, particularly the capacity to induce apatite formation, is essential for osteointegration at the tendon–bone interface [28]. Upon immersion in SBF for 7, 14 and 21 days, the functionalized sutures demonstrated a significant capacity for surface mineralization (Figure 3A). While the control PET surface remained inert, the Gel/TA/H-Ce sutures were rapidly covered by a dense layer of crystals. EDS analysis confirmed that these deposits were enriched in Ca and P, and FTIR spectra revealed phosphate-related absorption bands around 1030 and 560 cm^−1^, corresponding to P–O stretching and O–P–O bending vibrations, respectively, supporting the formation of apatite-like mineral deposits on the suture surfaces (Figure 3B) [29, 30]. This rapid *in vitro* biomineralization suggests that the Ce-MBGNs-coated sutures can establish a bioactive interface *in vivo*, potentially facilitating chemical bonding between the graft and native bone.

The successful fabrication of Ce-MBGNs and their stable integration onto PET sutures constitute essential prerequisites for subsequent biological regulation at the TBI. The uniform mesoporous architecture observed in this study is consistent with previous reports showing that MBGNs exhibit high specific surface area and ion-exchange capacity, which enhance bioactivity and cellular responsiveness [29, 31]. The Gel/TA matrix effectively anchors Ce-MBGNs onto the otherwise inert PET surface through noncovalent interactions, preserving the intrinsic redox activity of cerium while simultaneously improving surface hydrophilicity. Similar TA-based strategies have been reported to enhance coating stability and interfacial adhesion without compromising biofunctionality [32, 33]. Importantly, the dose-dependent mineralization behavior observed in SBF is consistent with prior studies suggesting that bioactive glass–mediated apatite formation may contribute to osteointegration at the TBI [34, 35]. Collectively, these findings demonstrate that the nanoengineered coating establishes a chemically active and osteoconductive interface, thereby providing a robust structural and biochemical foundation for subsequent immunomodulatory and regenerative effects.

### Cell viability and migration

BMSCs were characterized via flow cytometry, confirming >95% expression of positive markers (CD44, CD90) and <5% of negative markers (CD34, CD45) (Supplementary Figure S1A). Differentiation assays further validated their multipotency, with BMSCs adopting adipocyte, chondrocyte, and osteoblast phenotypes under induction (Supplementary Figure S1B–E).

**Figure 4:**
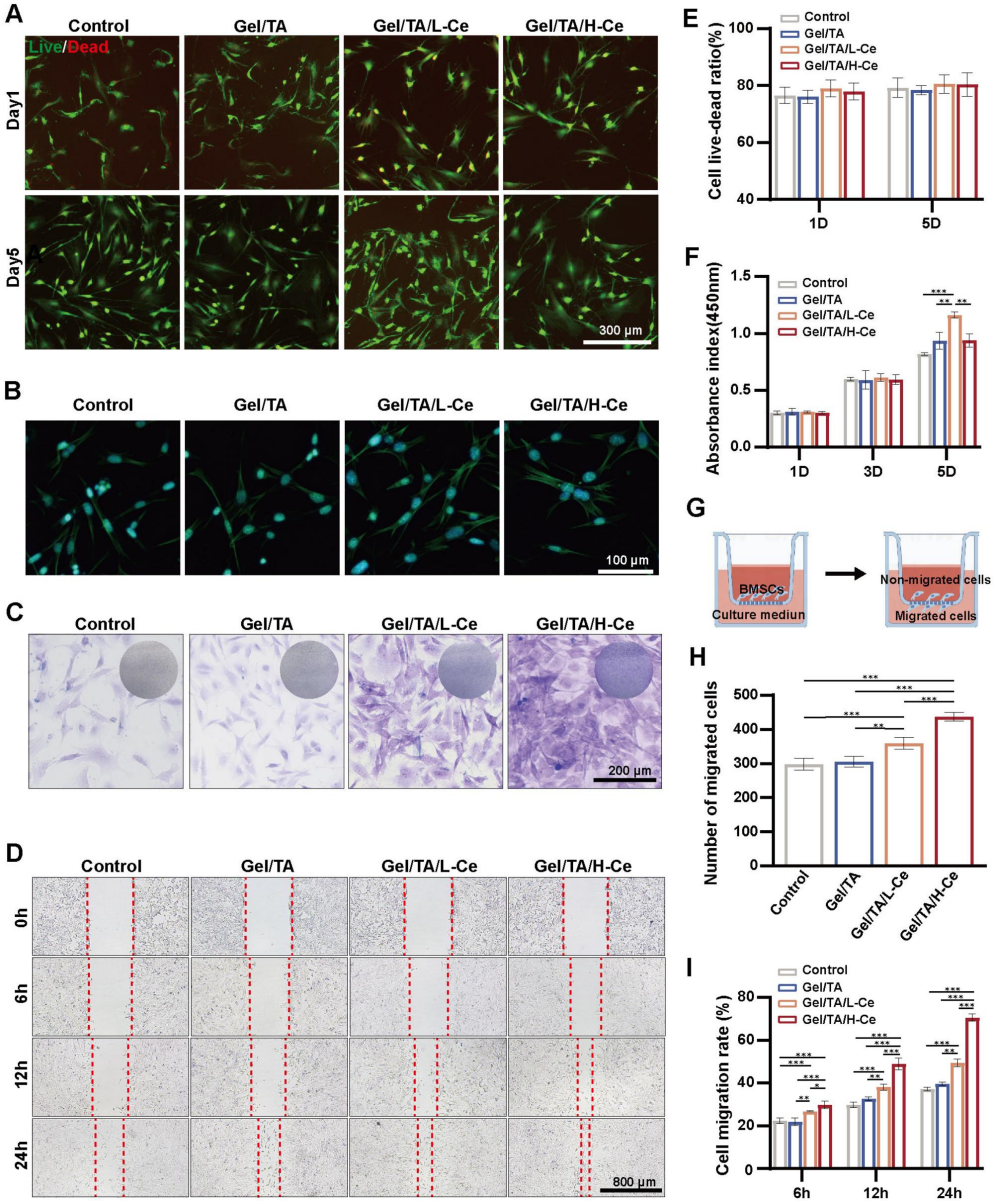
Cell viability, migration and invasion assay *in vitro*. (**A**) Fluorescent images of live and dead BMSCs cultured in various extract media. (**B**) Cytoskeleton staining of BMSCs seeded on control suture, and Gel/TA-, Gel/TA/L-Ce- and Gel/TA/H-Ce-coated suture after 3 days. (**C**) Transwell images and migration rates (**H**) of the cells cultured in different extract media. (**D**) Scratch assay images demonstrating cell migration, along with the corresponding quantitative data (**I**). (**E**) the live/dead ratio of BMSCs cultured in different extract media on days 1 and 5. (**F**) The absorbance indices of BMSCs cultured in different extract media on days 1, 3 and 5. (**G**) Schematic diagram of the transwell assay. *P < 0.05, **P < 0.01, ***P < 0.001.

Cytocompatibility of the coated sutures was assessed using live/dead staining and CCK-8 assays. Live/dead staining after 1 and 5 days of culture in suture extract media showed comparable BMSC viability across all groups, including the control (Figure 4A and E), underscoring the biocompatibility of Gel/TA/Ce-MBGNs/PET sutures. CCK-8 results (Figure 4F) indicated no significant viability differences on days 1 and 3; however, by day 5, the Gel/TA/L-Ce group exhibited a significantly higher optical density (1.16 ± 0.02) than the control, Gel/TA and Gel/TA/H-Ce groups, suggesting an optimal cerium concentration for proliferation.

Cytoskeletal and nuclear staining revealed enhanced BMSC adhesion on coated sutures (Figure 4B). Compared to the control, Gel/TA, Gel/TA/L-Ce and Gel/TA/H-Ce groups displayed increased pseudopod-like protrusions, indicative of improved cell–substrate interactions. Cell migration was evaluated via transwell and scratch assays. The Gel/TA/H-Ce group showed the highest migratory cell count in the transwell assay (430.67 ± 11.07) (Figure 4C–H) and the most significant cell migration rate (70.65 ± 1.54% after 24 h) (Figure 4D and I), highlighting a dose-dependent enhancement of migration by Ce-MBGNs.

The present findings demonstrate that Gel/TA/Ce-MBGN–modified sutures create a cytocompatible microenvironment that supports early cellular processes essential for TBI regeneration. Across all experimental groups, BMSCs maintained high viability, indicating that neither the Gel/TA coating nor the incorporation of Ce-MBGNs exerted cytotoxic effects. The increased proliferative activity observed at moderate Ce concentrations suggests an optimal balance between material bioactivity and cellular metabolic demand. Notably, while L-Ce promotes BMSC proliferation more effectively, the H-Ce group exhibits superior effects on cell migration, osteogenic/chondrogenic differentiation and macrophage immunomodulation. This indicates that the optimal Ce-MBGN dose may vary depending on the specific cellular function assessed, with lower doses favoring proliferation and higher doses enhancing multi-lineage differentiation and immunoregulatory outcomes.

These findings are consistent with previous studies demonstrating that surface-modified PET materials enhance cell–material interactions without compromising biocompatibility [36, 37]. Furthermore, the observed enhancement of BMSC migration is consistent with reports indicating that ionic cues released by bioactive glasses promote mesenchymal stem cell motility and cytoskeletal organization [38, 39]. Such enhanced cell recruitment has been directly associated with improved fibrocartilage formation and tendon–bone integration in rotator cuff repair models [40, 41], thereby supporting the biological relevance of the present findings.

### *In vitro* osteogenic differentiation

**Figure 5:**
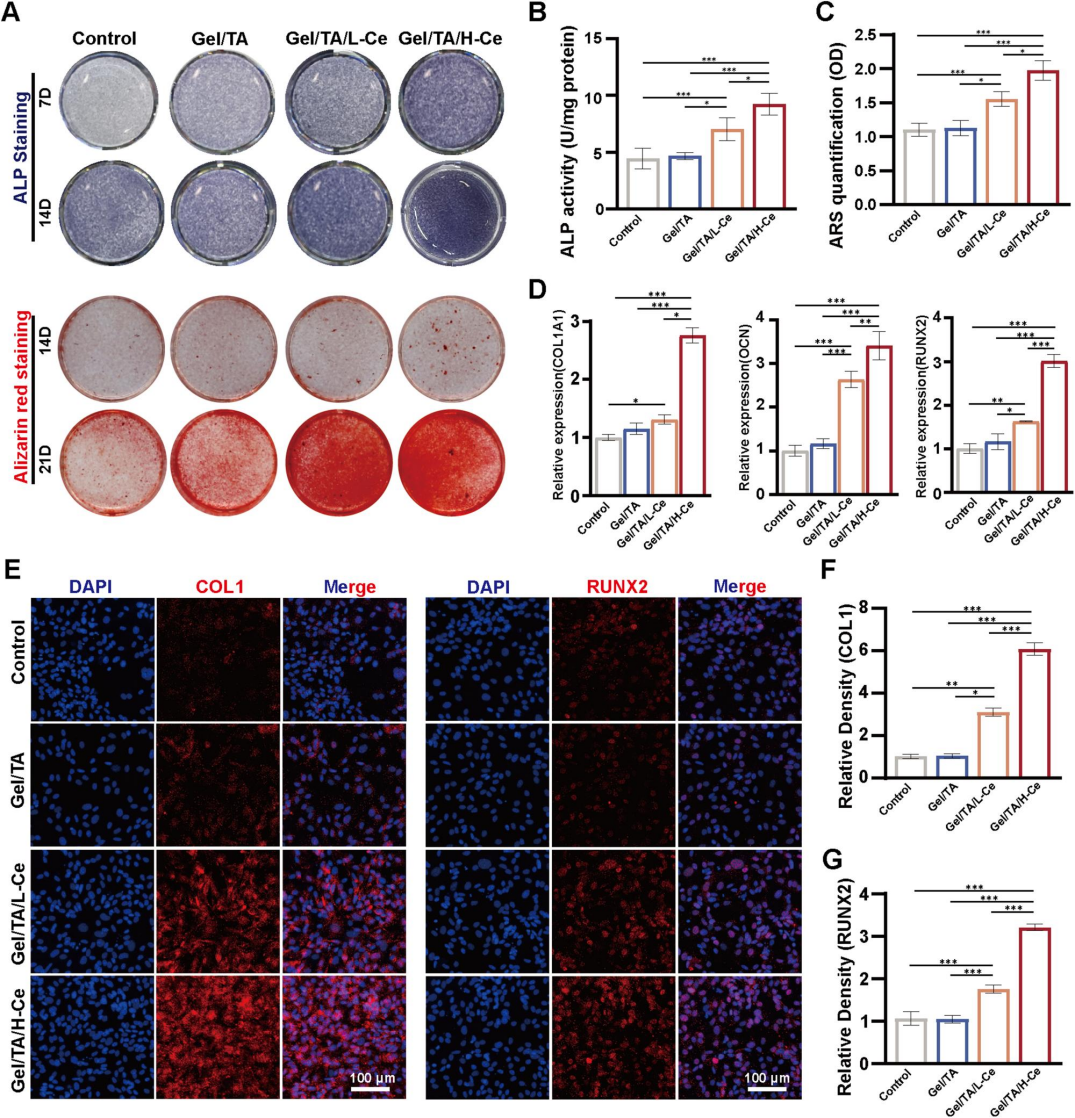
The osteogenic effect of Gel/TA/Ce-MBGNs/PET sutures *in vitro*. (**A**) ALP staining images on days 7 and 14 and ARS staining images on days 14 and 21. (**B**) ALP activity after 7 days. (**C**) Quantification of cellular mineralization. (**D**) RT-qPCR measurements of COL1A1, OCN and RUNX2 gene expression in BMSCs after 7 days of osteogenic induction with osteogenic extract induction medium. (**E**) Immunofluorescence staining of COL1 and RUNX2 in BMSCs after 7 days of osteogenic induction with osteogenic extract induction medium and associated quantification. (**F**, **G**) Immunofluorescence quantification of COL1 and RUNX2. *P < 0.05, **P < 0.01, ***P < 0.001.

The osteogenic potential of the coated sutures was investigated by culturing BMSCs in osteogenic induction media. ALP staining and activity assays on days 7 and 14, alongside ARS staining on days 14 and 21, demonstrated superior osteogenic induction in the Gel/TA/H-Ce group (Figure 5A–C). ALP activity and ARS absorbance (1.98 ± 0.12) were significantly elevated, reflecting enhanced early osteogenic commitment and mineral deposition, respectively. To further investigate the osteogenic potential, RT-qPCR was used to assess the expression of key osteogenic markers after 7 days of culture in osteogenic extract induction medium. The expression levels of osteogenic markers—COL1A1 (2.75 ± 0.11), OCN (3.42 ± 0.26) and RUNX2 (3.01 ± 0.12)—were significantly upregulated in the Gel/TA/H-Ce group (Figure 5D). Immunofluorescence staining confirmed higher COL1 and RUNX2 protein levels, with fluorescence intensity in the Gel/TA/H-Ce group surpassing that in Gel/TA/L-Ce, Gel/TA and control groups (Figure 5E–G). These data collectively indicate that Ce-MBGN incorporation enhances osteogenic differentiation in a concentration-dependent manner, with minimal differences between the Gel/TA and control groups.

The osteogenic findings demonstrate that the incorporation of Ce-MBGNs markedly enhances the osteoinductive capacity of PET sutures, with the Gel/TA/H-Ce group exhibiting the highest alkaline phosphatase activity, mineral deposition and expression of osteogenic markers. The observed dose-dependent response indicates that sustained ionic signaling from Ce-MBGNs, rather than the Gel/TA matrix alone, serves as the primary driver of osteogenic differentiation. This behavior is consistent with previous studies showing that silicon and calcium ions released from bioactive glasses activate osteogenic transcriptional programs in mesenchymal stem cells [42–44]. This incorporation has been shown to mitigate oxidative stress in osteogenic environments, which may help sustain osteogenic signaling under conditions that would otherwise impair differentiation [45]. Given that inadequate bone regeneration is a well-recognized contributor to tendon–bone repair failure, the enhanced osteogenic response observed in this study is of substantial relevance to functional enthesis reconstruction [46, 47].

### *In vitro* chondrogenic differentiation

**Figure 6:**
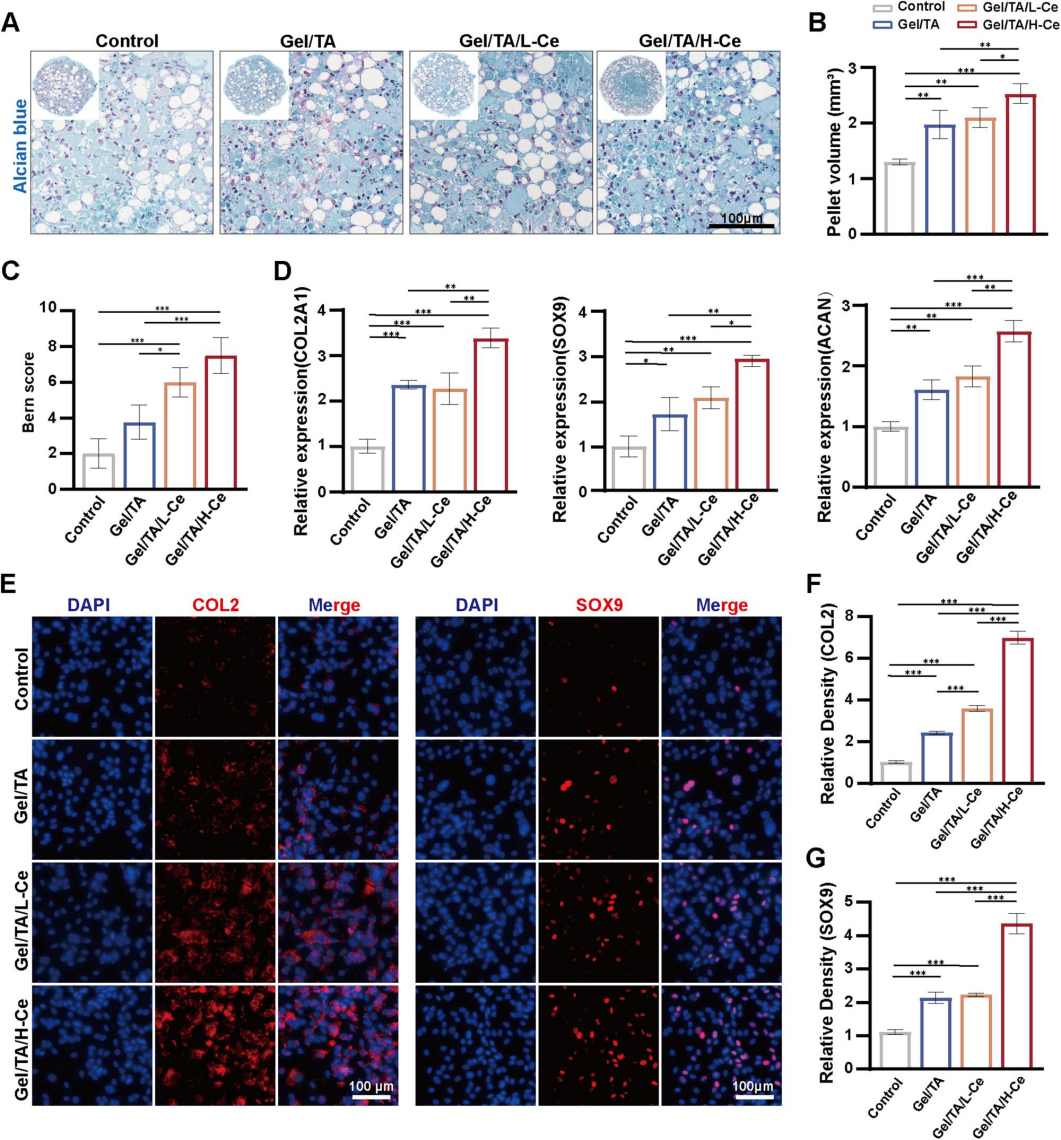
The chondrogenic effect of Gel/TA/Ce-MBGNs/PET sutures *in vitro*. (**A**) Alcian blue staining images of pellets on day 21. (**B**) Volumes of the pellets cultured with different chondrogenic extract induction media. (**C**) The Bern scores for cartilage pellets. (**D**) RT-qPCR measurements of COL2A1, SOX9 and ACAN gene expression in BMSCs after 7 days of chondrogenic induction with chondrogenic extract induction medium. (**E**) Immunofluorescence staining of COL2 and SOX9 in BMSCs after 7 days of chondrogenic induction with chondrogenic extract induction medium. (**F**, **G**) Immunofluorescence quantification of COL2 and SOX9. *P < 0.05, **P < 0.01, ***P < 0.001.

To assess the chondrogenic potential of PET sutures modified with various coatings, we induced BMSCs toward chondrogenesis using both microsphere and planar culture systems in a chondrogenic induction medium supplemented with extracts from the coated sutures. This dual approach enabled us to evaluate how surface modifications influence BMSC differentiation, a critical factor in cartilage repair and tissue engineering applications. After 21 days, Alcian blue staining of proteoglycan-rich extracellular matrix (ECM) revealed pronounced differences among the groups (Figure 6A). Microspheres from the Gel/TA/H-Ce group, featuring a Gel/TA coating with high Ce-MBGNs, exhibited the most intense staining, indicative of enhanced proteoglycan synthesis. Quantitative analysis of cartilage particle volume further confirmed these observations: the Gel/TA/H-Ce group yielded a significantly larger volume (2.53 ± 0.16 mm³) compared to Gel/TA/L-Ce (2.10 ± 0.15 mm³), Gel/TA (1.98 ± 0.22 mm³) and uncoated control groups (1.30 ± 0.05 mm³) (Figure 6B). Histological scoring using the Bern scale corroborated these findings, with the Gel/TA/H-Ce group demonstrating superior cartilage pellet quality (Figure 6C).

RT-qPCR revealed significant upregulation of chondrogenic marker genes—COL2A1, SOX9 and ACAN—in the Gel/TA/H-Ce group, with expression levels reaching 3.38 ± 0.18, 2.91 ± 0.10 and 2.57 ± 0.14, respectively (Figure 6D). These values markedly exceeded those in the Gel/TA/L-Ce (2.28 ± 0.29, 2.09 ± 0.20, 1.83 ± 0.14), Gel/TA (2.36 ± 0.08, 1.73 ± 0.30, 1.61 ± 0.13) and control groups (1.01 ± 0.13, 1.02 ± 0.19, 1.00 ± 0.06). Immunofluorescence staining for COL2 and SOX9 on day 7 further supported these results, with the Gel/TA/H-Ce group displaying the strongest fluorescence signals, quantitatively validated by intensity measurements (Figure 6E–G). These data suggest that the synergistic effects of Gel/TA and high-dose Ce-MBGNs enhance chondrogenic differentiation, likely through improved ECM production and transcriptional activation of cartilage-specific pathways.

The chondrogenic assays indicate that the incorporation of Ce-MBGNs substantially enhances the cartilage-like differentiation of BMSCs, with the Gel/TA/H-Ce group exhibiting the most pronounced proteoglycan deposition and upregulation of chondrogenic markers. The coordinated increases in COL2A1, SOX9 and ACAN expression suggest that the nanoengineered interface supports both early chondrogenic commitment and subsequent ECM maturation. Although the Gel/TA coating alone modestly promoted chondrogenesis, the presence of Ce-MBGNs clearly amplified this effect, indicating a synergistic contribution from sustained ionic signaling. These findings are consistent with previous reports demonstrating that ions released from bioactive glasses stimulate chondrogenic differentiation and matrix synthesis in mesenchymal stem cells [48]. In the context of tendon–bone healing, enhanced chondrogenesis is particularly relevant, as fibrocartilage formation is a critical component of functional enthesis regeneration [49]. Collectively, these results suggest that Ce-MBGNs-functionalized sutures provide biochemical cues conducive to fibrocartilaginous interface formation.

### Immunomodulatory activity *in vitro*

**Figure 7:**
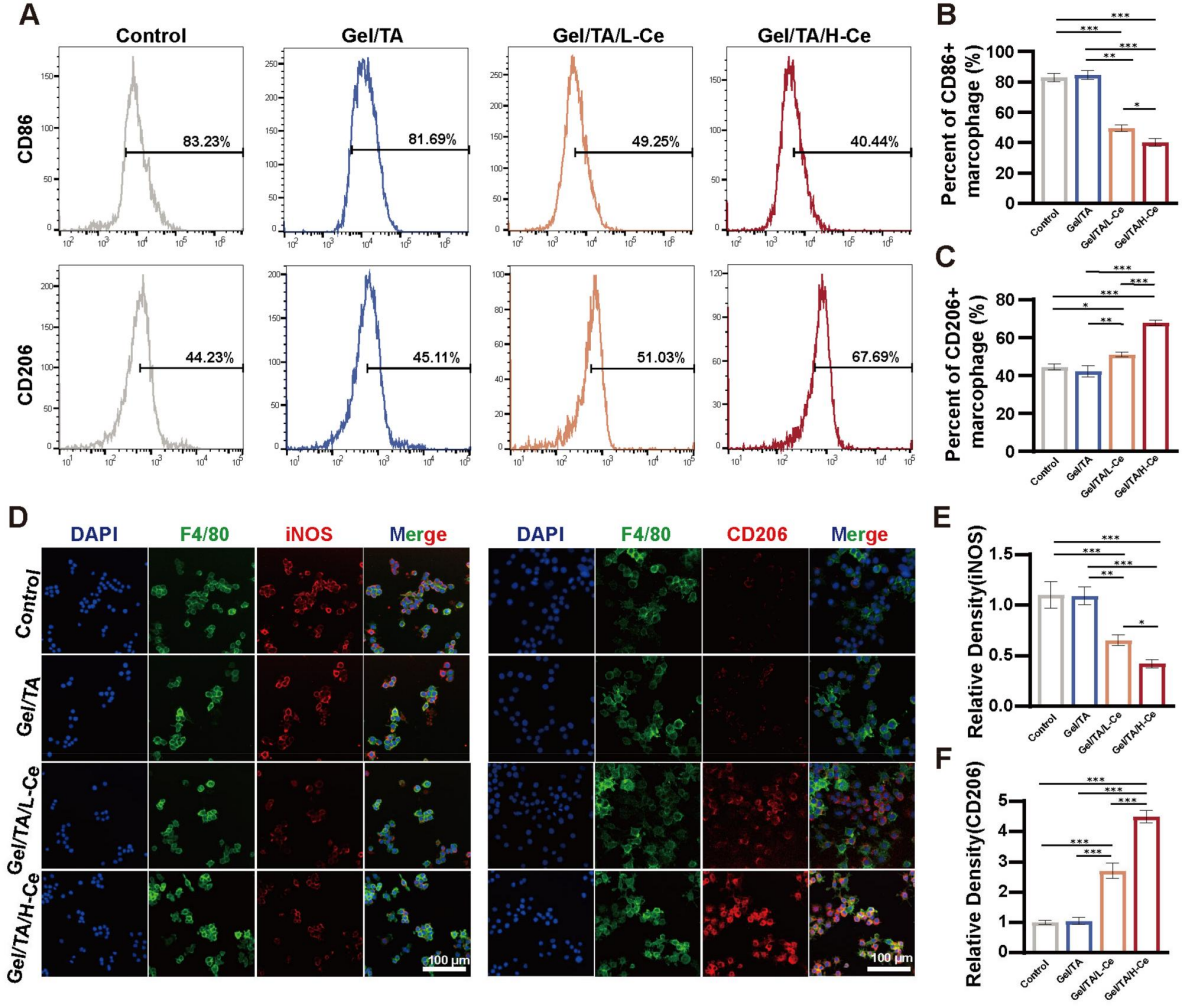
Immunomodulatory activity *in vitro*. (**A**) Flow cytometry of CD86-positive M1 macrophages, and CD206-positive M2 macrophages, and corresponding quantification (**B**, **C**). (**D**) immunofluorescence of iNOS in RAW264.7 cells after LPS stimulation and immunofluorescence of CD206 in RAW264.7 cells following incubation with IL-4 and IL-13. (**E**, **F**) Immunofluorescence quantification of iNOS and CD206. *P < 0.05, **P < 0.01, ***P < 0.001.

Macrophage polarization plays a pivotal role in shaping the immune microenvironment during tissue repair, particularly at the tendon–bone interface. To explore the immunomodulatory capacity of the Gel/TA/H-Ce coating, we analyzed RAW264.7 macrophage responses using flow cytometry. The Gel/TA/H-Ce group exhibited a significant reduction in CD86-positive (M1) macrophages and a concurrent increase in CD206-positive (M2) macrophages compared to controls (Figure 7A–C). In contrast, the Gel/TA and control groups showed no notable shifts in polarization, indicating that Ce-MBGNs are essential for driving M2 polarization. Immunofluorescence staining for iNOS (M1 marker) and CD206 (M2 marker), using F4/80 as a pan-macrophage identifier, reinforced these findings (Figure 7D). The Gel/TA/H-Ce group displayed reduced iNOS fluorescence intensity (0.42 ± 0.03) and elevated CD206 intensity (4.49 ± 0.16) relative to other groups (Figure 7E and F). These results highlight the coating’s ability to skew macrophage behavior toward an anti-inflammatory, pro-regenerative M2 phenotype, a key mechanism for enhancing TBI healing.

The immunomodulatory assays demonstrate that Ce-MBGNs-functionalized sutures effectively regulate macrophage polarization, favoring a shift toward the anti-inflammatory M2 phenotype. Compared with the control and Gel/TA groups, the Gel/TA/H-Ce coating significantly reduced the expression of M1-associated markers while enhancing CD206 expression, indicating that the nanoengineered interface actively modulates macrophage behavior rather than merely supporting passive cell survival. This regulatory effect is particularly relevant at the TBI, where macrophages are persistently exposed to mechanical loading and oxidative byproducts following repair [50]. In this context, the preferential M2 polarization induced by the Ce-MBGNs interface suggests that local modulation of the redox and ionic microenvironment can bias macrophage responses toward a reparative phenotype without fully suppressing early inflammatory signaling. Comparable interface-driven immunomodulatory effects have been reported in bioactive glass–based systems [51, 52], supporting the concept that material-mediated microenvironmental regulation can shape immune dynamics during musculoskeletal regeneration. Collectively, these findings suggest that Ce-MBGN coatings contribute to the regulation of macrophage behavior at the TBI, thereby supporting a local immune environment favorable for interface repair.

### *In vitro* antioxidant activity

**Figure 8:**
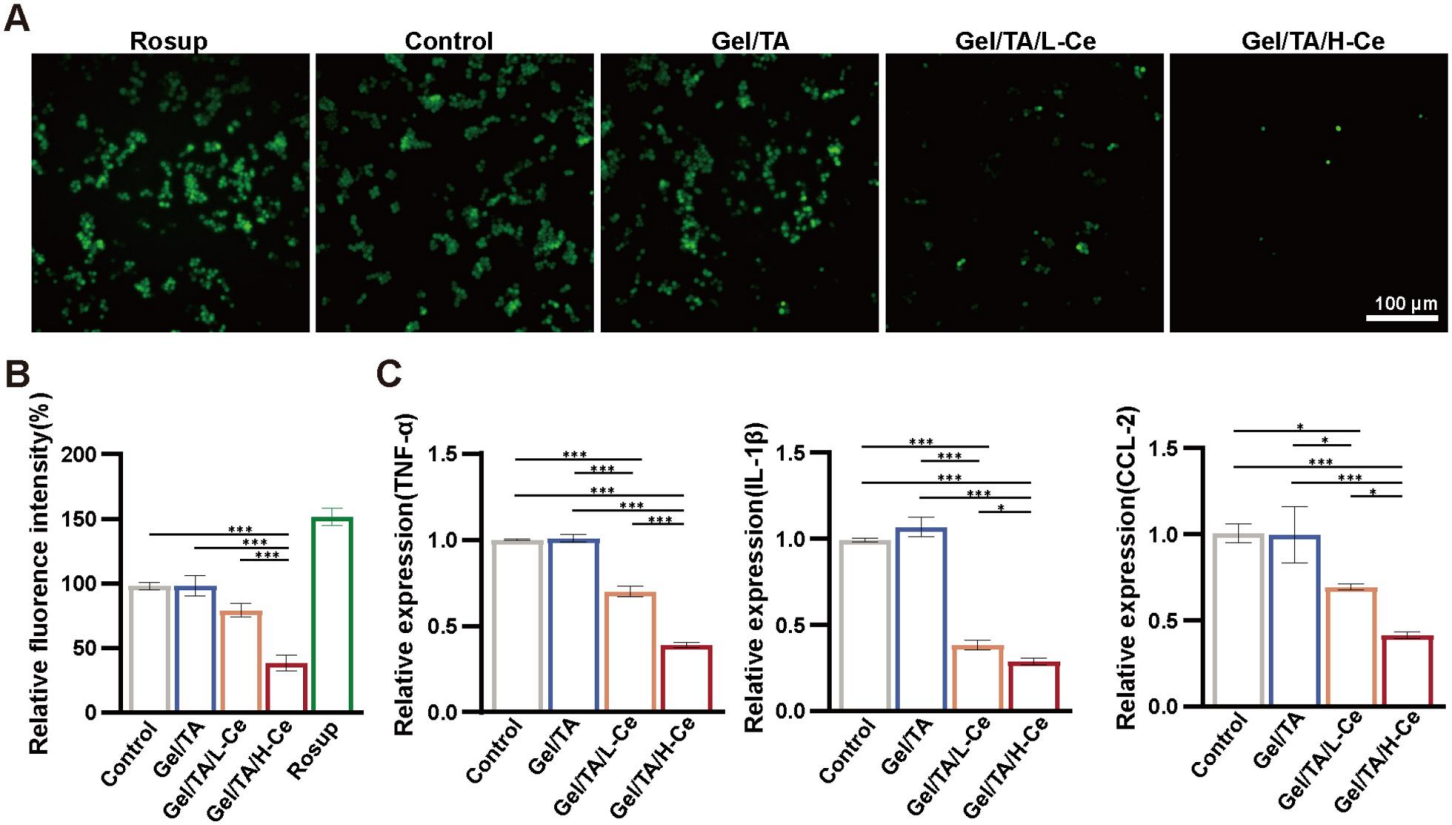
Antioxidant activity of Gel/TA/Ce-MBGNs/PET sutures. (**A**) Fluorescence images and (**B**) quantification of relative ROS fluorescence intensity in RAW264.7 macrophages using a ROS assay kit. (**C**) RT-qPCR analysis of TNF-α, IL-1β and CCL-2 expression in LPS-stimulated RAW264.7 macrophages cultured with different suture extract media. *P < 0.05, **P < 0.01, ***P < 0.001.

ROS during tendon–bone healing exacerbates inflammation and impairs regeneration. We evaluated the antioxidant properties of the Gel/TA/H-Ce coating by measuring ROS levels in LPS-stimulated RAW264.7 cells. Fluorescence imaging revealed a significant reduction in ROS in the Gel/TA/H-Ce group, with an approximately 61% decrease in intensity (38.31 ± 4.81%) compared to controls (98.01 ± 2.38%) (Figure 8A and B). The Gel/TA group showed no substantial difference from controls, underscoring the role of Ce-MBGNs in ROS scavenging. RT-qPCR analysis of pro-inflammatory genes (TNF-α, IL-1β and CCL-2) in M1 macrophages further demonstrated that the Gel/TA/H-Ce group suppressed expression to 0.39 ± 0.01, 0.29 ± 0.02 and 0.41 ± 0.02, respectively, compared to controls (Figure 8C). This dual antioxidant and anti-inflammatory effect is likely mediated by the redox activity of Ce-MBGNs, which establishes a regenerative microenvironment and may facilitate the reprogramming of macrophages from the M1 to the M2 phenotype.

The antioxidant results indicate that Ce-MBGNs-functionalized sutures effectively suppress excessive intracellular ROS accumulation in activated macrophages, accompanied by reduced expression of pro-inflammatory genes. Notably, this effect was observed under inflammatory stimulation rather than basal conditions, suggesting that the coating functions in a context-dependent manner rather than indiscriminately scavenging ROS. Such localized redox regulation is particularly relevant at the TBI, where persistent oxidative stress has been associated with sustained macrophage activation and impaired resolution of inflammation. Consistent with the overarching framework of this study, attenuation of ROS at the material–tissue interface may help constrain downstream pathways linked to inflammatory persistence, such as aberrant HIF-1α signaling, thereby promoting stabilization of macrophage phenotypes during early healing [53]. Collectively, these findings indicate that Ce-MBGN coatings provide functional redox control at the interface level, supporting immune regulation under oxidative challenge rather than simply eliminating ROS.

### *In vivo* animal study

**Figure 9:**
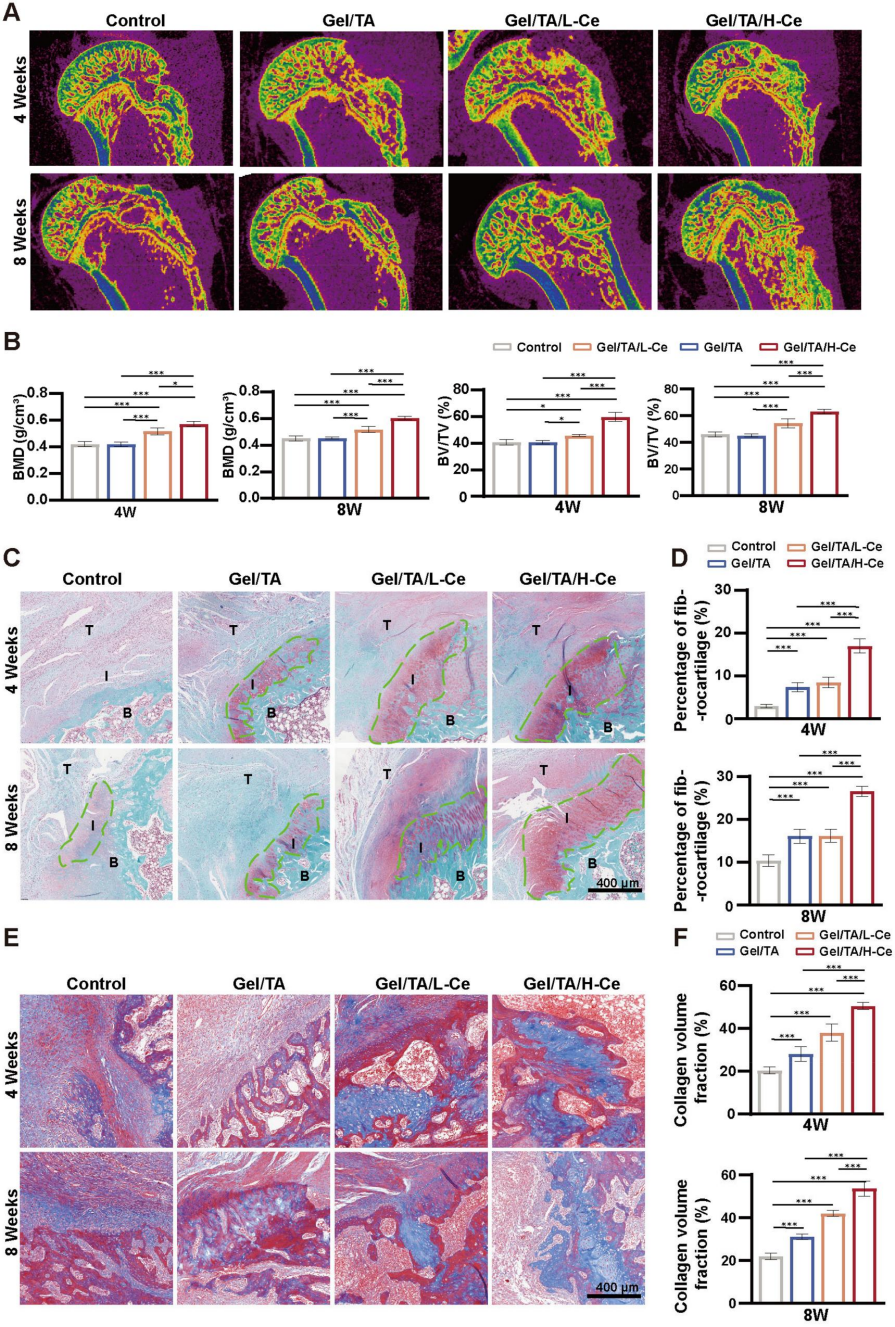
Micro-CT and histological analyses *in vivo*. (**A**) Micro-CT images of the cross-sectional view of the humeral head after 4 and 8 weeks. (**B**) BMD and BV/TV values of the TBI. (**C**) Safranin O/fast green staining of the TBI. (**D**) Percentages of fibrocartilage in the control, Gel/TA, Gel/TA/L-Ce and Gel/TA/H-Ce groups. (**E**) Masson’s trichrome staining of the TBI. (**F**) Quantitative analysis of the collagen area fraction in the control, Gel/TA, Gel/TA/L-Ce and Gel/TA/H-Ce groups. T, tendon, I, tendon–bone interface, B, bone. *P < 0.05, **P < 0.01, ***P < 0.001.

To validate these *in vitro* findings, we established a rat RCT model using coated PET sutures, with uncoated sutures as controls. Micro-CT analysis of the greater tuberosity revealed enhanced bone regeneration in the Gel/TA/H-Ce group at 4 and 8 weeks post-surgery (Figure 9A). BMD measurements showed significantly higher values in the Gel/TA/H-Ce group compared to other groups (Figure 9B). Safranin O/Fast Green staining of the TBI demonstrated progressive fibrocartilage formation, with the Gel/TA/H-Ce group exhibiting a larger fibrocartilage area (26.42 ± 0.93%) by week 8 (Figure 9C and D). Masson’s trichrome staining was then used to further evaluate collagen deposition and tendon–bone interface regeneration. The Gel/TA/L-Ce and Gel/TA/H-Ce groups showed increased collagen content and more organized fiber arrangement (Figure 9E and F). H&E staining revealed reduced inflammatory infiltration and more organized collagen fibers in the Gel/TA/H-Ce group, with tendon maturity scores of 7.75 ± 1.09 at 4 weeks and 9.5 ± 1.80 at 8 weeks (Supplementary Figure S2A and B).

**Figure 10:**
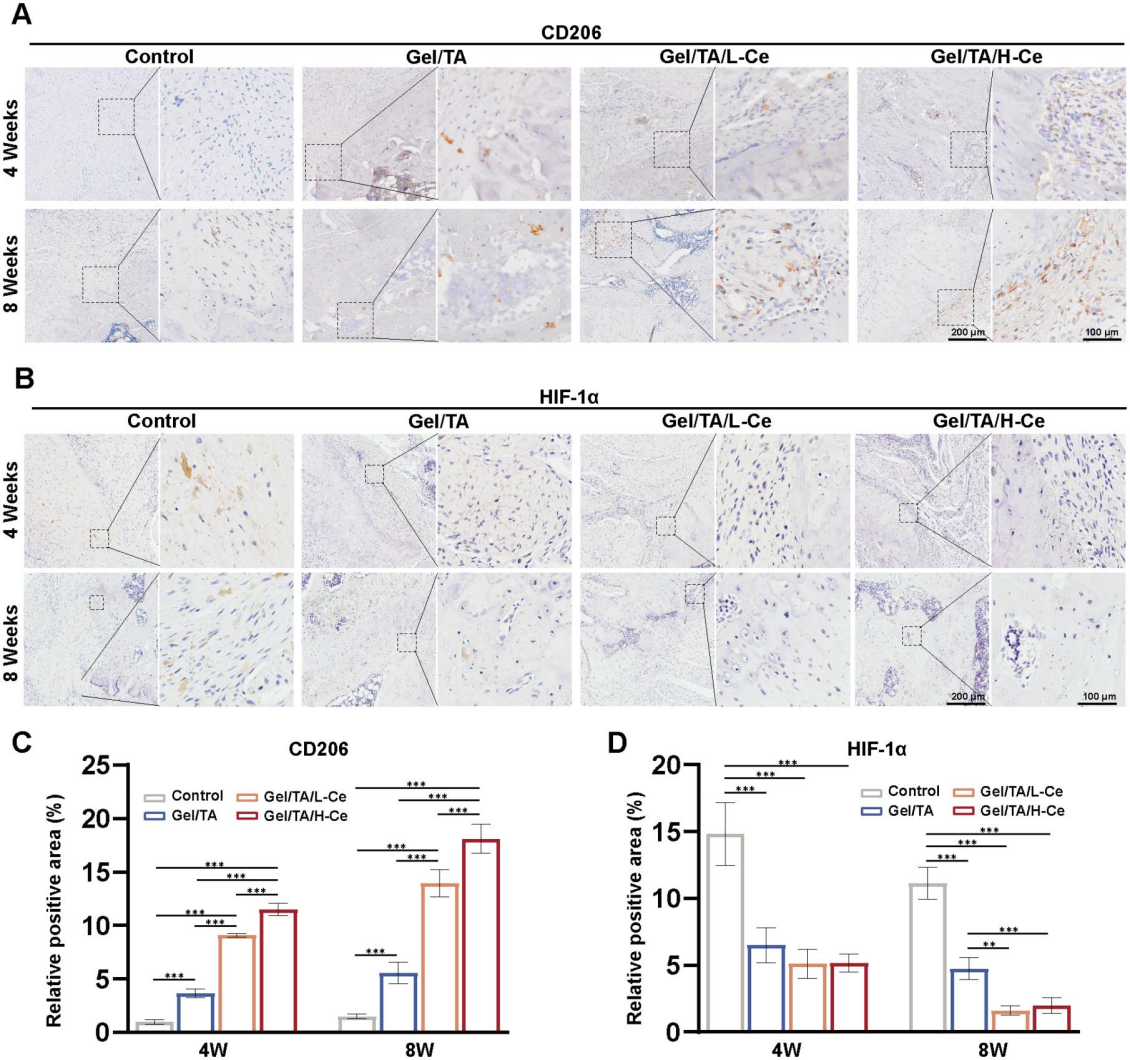
Immunohistochemical staining of newly formed TBI tissues. (**A**) Immunohistochemical staining of CD206 at the TBI and associated expression levels at 4 and 8 weeks after treatment (**C**). (**B**) Immunohistochemical staining of HIF-1α at the TBI and associated expression levels 4 and 8 weeks after the treatment (**D**). *P < 0.05, **P < 0.01, ***P < 0.001.

Immunohistochemical analysis elucidated the underlying mechanisms. The Gel/TA/H-Ce group displayed a higher proportion of CD206-positive M2 macrophages (11.50 ± 0.51% at 4 weeks; 18.10 ± 1.19% at 8 weeks) than other groups (Figure 10A and C), indicating sustained immunomodulation *in vivo*. Staining for HIF-1α, a regulator of ROS-mediated inflammation, showed reduced expression in the Gel/TA/H-Ce group (5.16 ± 0.59% at 4 weeks; 1.99 ± 0.50% at 8 weeks) compared to controls (14.82 ± 2.04% at 4 weeks) (Figure 10B and D). These findings suggest that the Gel/TA/H-Ce coating enhances TBI repair by integrating chondrogenic, immunomodulatory and antioxidant effects, which may be associated with ROS/HIF-1α-mediated macrophage reprogramming.

The *in vivo* outcomes suggest that the therapeutic benefits of Ce-MBGNs-functionalized sutures do not arise from isolated enhancement of a single tissue component, but rather from coordinated regulation of the tendon–bone healing microenvironment. The concurrent improvements in bone formation, fibrocartilage continuity and collagen organization indicate that the interface progressed toward a more native-like enthesis architecture rather than a scar-mediated attachment. We propose that this strategy acts through a multi-stage cascade: it primarily “sets the stage” through early modulation of the inflammatory microenvironment via ROS scavenging and M2 macrophage polarization. Subsequently, the sustained release of bioactive ions (e.g. Si, Ca and Ce) from the mesoporous network likely continues to exert beneficial osteoconductive and chondrogenic effects throughout the later remodeling stages of the repair process. Crucially, these structural improvements were accompanied by increased local M2 macrophage prevalence and reduced HIF-1α expression, suggesting that immune regulation and redox homeostasis are closely linked to improved healing outcomes rather than representing isolated phenomena. Previous studies in rotator cuff repair have demonstrated that interventions targeting osteogenesis, fibrocartilage formation or macrophage polarization individually can enhance tendon–bone healing [54]. The present findings extend this body of work by showing that a single interface-engineered suture can concurrently modulate multiple regenerative processes *in vivo*, underscoring the significance of localized, material-driven regulation for functional enthesis reconstruction.

## Conclusions

In this study, we developed a nanoengineered PET suture interface by incorporating Ce-MBGNs into a Gel/TA coating to address the immunological and oxidative barriers that impede TBI regeneration. The resulting Gel/TA/Ce-MBGN coating establishes a multifunctional biointerface with robust ROS–scavenging and immunomodulatory capabilities.

The functionalized sutures exhibit excellent cytocompatibility and actively promote osteogenic and chondrogenic differentiation of mesenchymal stem cells *in vitro*, thereby supporting the formation of both bone and fibrocartilage components essential for native enthesis reconstruction. Concurrently, Ce-MBGN incorporation effectively reprograms macrophage polarization toward a pro-regenerative M2 phenotype by attenuating intracellular ROS accumulation. *In vivo*, these combined effects translate into enhanced fibrocartilage formation, improved bone quality and more organized tendon–bone integration in a rat rotator cuff repair model. The reduced expression of HIF-1α at the repair site further indicates that localized redox regulation plays a critical role in restoring macrophage immunometabolic balance during healing.

Overall, this work highlights the therapeutic potential of immunoregulatory and antioxidant suture interface engineering as a targeted surface-functionalization strategy to coordinate immune responses and multi-tissue regeneration at the TBI. By integrating ROS modulation with osteo-chondrogenic bioactivity into a pre-existing, clinically relevant suture platform, this approach offers a promising and translatable solution for improving the long-term success of rotator cuff repair.

## Supplementary Material

rbag121_Supplementary_Data

## Data Availability

The data that support the findings of this study are available from the corresponding author upon reasonable request.

## References

[1] Zhao S , Su W, Shah V, Hobson D, Yildirimer L, Yeung KWK, Zhao J, Cui W, Zhao X. Biomaterials based strategies for rotator cuff repair. Colloids Surf B Biointerfaces 2017;157:407–16. 28633121 10.1016/j.colsurfb.2017.06.004

[2] Miller BS , Downie BK, Kohen RB, Kijek T, Lesniak B, Jacobson JA, Hughes RE, Carpenter JE. When do rotator cuff repairs fail? Serial ultrasound examination after arthroscopic repair of large and massive rotator cuff tears. Am J Sports Med 2011;39:2064–70. 21737833 10.1177/0363546511413372

[3] Abdalla AA , Pendegrass CJ. Biological approaches to the repair and regeneration of the rotator cuff tendon-bone enthesis: a literature review. Biomater Transl 2023;4:85–103. 38283917 10.12336/biomatertransl.2023.02.004 PMC10817785

[4] Huegel J , Williams AA, Soslowsky LJ. Rotator cuff biology and biomechanics: a review of normal and pathological conditions. Curr Rheumatol Rep 2015;17:476. 25475598 10.1007/s11926-014-0476-x

[5] Akbar M , MacDonald L, Crowe LAN, Carlberg K, Kurowska-Stolarska M, Ståhl PL, Snelling SJB, McInnes IB, Millar NL. Single cell and spatial transcriptomics in human tendon disease indicate dysregulated immune homeostasis. Ann Rheum Dis 2021;80:1494–7. 34001518 10.1136/annrheumdis-2021-220256 PMC8522454

[6] Chen Z , Jin M, He H, Dong J, Li J, Nie J, Wang Z, Xu J, Wu F. Mesenchymal stem cells and macrophages and their interactions in tendon-bone healing. J Orthop Translat 2023;39:63–73. 37188000 10.1016/j.jot.2022.12.005 PMC10175706

[7] Zhang X , Song W, Liu Y, Han K, Wu Y, Cho E, Fang Z, Jiang L, Hu Y, Zhu X, Jiang J, Huangfu X, Zhao J. Healthy tendon stem Cell-Derived exosomes promote tendon-to-bone healing of aged chronic rotator cuff tears by breaking the positive-feedback cross-talk between senescent tendon stem cells and macrophages through the modulation of macrophage polarization. Small 2024; 20: e2311033. 38459643 10.1002/smll.202311033

[8] Benson RT , McDonnell SM, Knowles HJ, Rees JL, Carr AJ, Hulley PA. Tendinopathy and tears of the rotator cuff are associated with hypoxia and apoptosis. J Bone Joint Surg Br 2010;92:448–53. 20190320 10.1302/0301-620X.92B3.23074 PMC2843163

[9] Cortés M , Brischetto A, Martinez-Campanario MC, Ninfali C, Domínguez V, Fernández S, Celis R, Esteve-Codina A, Lozano JJ, Sidorova J, Garrabou G, Siegert AM, Enrich C, Pintado B, Morales-Ruiz M, Castro P, Cañete JD, Postigo A. Inflammatory macrophages reprogram to immunosuppression by reducing mitochondrial translation. Nat Commun 2023;14:7471. 37978290 10.1038/s41467-023-42277-4 PMC10656499

[10] Li Y , Liang Q, Zhou L, Cao Y, Yang J, Li J, Liu J, Bi J, Liu Y. An ROS-responsive artesunate prodrug nanosystem co-delivers dexamethasone for rheumatoid arthritis treatment through the HIF-1α/NF-κB Cascade regulation of ROS scavenging and macrophage repolarization. Acta Biomater 2022;152:406–24. 36055613 10.1016/j.actbio.2022.08.054

[11] Wu KK , Xu X, Wu M, Li X, Hoque M, Li GHY, Lian Q, Long K, Zhou T, Piao H, Xu A, Hui HX, Cheng KK. MDM2 induces pro-inflammatory and glycolytic responses in M1 macrophages by integrating iNOS-nitric oxide and HIF-1α pathways in mice. Nat Commun 2024;15:8624. 39366973 10.1038/s41467-024-53006-w PMC11452520

[12] Yu C , Feng S, Li Y, Chen J. Application of nondegradable synthetic materials for tendon and ligament injury. Macromol Biosci 2023; 23: e2300259. 37440424 10.1002/mabi.202300259

[13] Taha ME , Schneider K, Clarke EC, O’Briain DE, Smith MM, Cunningham G, Cass B, Young AA. A biomechanical comparison of different suture materials used for arthroscopic shoulder procedures. Arthroscopy 2020;36:708–13. 31727418 10.1016/j.arthro.2019.08.048

[14] Wang S , Xu C, Yu Y, Li J, Chen T, Wang J, Liu C. Immunoregulative coating for scarless healing in anterior cruciate ligament reconstruction. Bioact Mater 2025;45:71–87. 39624422 10.1016/j.bioactmat.2024.11.007 PMC11609481

[15] Shang P , Xiang Y, Xing C, Chen S, Yuan F. Procyanidin-crosslinked gradient silk fibroin composite nanofiber scaffold with sandwich structure for rotator cuff repair. Biomater Adv 2025;169:214183. 39813740 10.1016/j.bioadv.2025.214183

[16] Wang S , Ge Y, Ai C, Jiang J, Cai J, Sheng D, Wan F, Liu X, Hao Y, Chen J, Chen S. Enhance the biocompatibility and osseointegration of polyethylene terephthalate ligament by plasma spraying with hydroxyapatite in vitro and in vivo. Int J Nanomedicine 2018;13:3609–23. 29983557 10.2147/IJN.S162466 PMC6026588

[17] Ramachandran B , Chakraborty S, Kannan R, Dixit M, Muthuvijayan V. Immobilization of hyaluronic acid from Lactococcus lactis on polyethylene terephthalate for improved biocompatibility and drug release. Carbohydr Polym 2019;206:132–40. 30553306 10.1016/j.carbpol.2018.10.099

[18] Zheng K , Niu W, Lei B, Boccaccini AR. Immunomodulatory bioactive glasses for tissue regeneration. Acta Biomater 2021;133:168–86. 34418539 10.1016/j.actbio.2021.08.023

[19] Zheng K , Sui B, Ilyas K, Boccaccini AR. Porous bioactive glass micro- and nanospheres with controlled morphology: developments, properties and emerging biomedical applications. Mater Horiz 2021;8:300–35. 34821257 10.1039/d0mh01498b

[20] Gong Z , Chen Z, Li D, Lu X, Wu J, Sun H, Wang X, Liu S, Xia X, Lu F, Jiang J, Sun C, Wang H, Zeng F, Ma X. Hydrogel loaded with cerium-manganese nanoparticles and nerve growth factor enhances spinal cord injury repair by modulating immune microenvironment and promoting neuronal regeneration. J Nanobiotechnology 2025;23:29. 39833803 10.1186/s12951-025-03098-3 PMC11748312

[21] Cai J , Zhang L, Chen J, Chen S. Silk fibroin coating through EDC/NHS crosslink is an effective method to promote graft remodeling of a polyethylene terephthalate artificial ligament. J Biomater Appl 2019;33:1407–14. 30885033 10.1177/0885328219836625

[22] Shi S , Fan W, Tao R, Xu H, Lu Y, Han F, Yang S, Zhou X, Zhou Z, Wan F. Natural biomineralization-inspired magnesium silicate composite coating upregulates osteogenesis, enabling strong anterior cruciate ligament graft-bone healing in vivo. ACS Biomater Sci Eng 2021;7:133–43. 33332969 10.1021/acsbiomaterials.0c01441

[23] Zhong G , Qiu M, Zhang J, Jiang F, Yue X, Huang C, Zhao S, Zeng R, Zhang C, Qu Y. Fabrication and characterization of PVA@PLA electrospinning nanofibers embedded with bletilla striata polysaccharide and rosmarinic acid to promote wound healing. Int J Biol Macromol 2023;234:123693. 36806778 10.1016/j.ijbiomac.2023.123693

[24] Fu J , Hui Q, Gu YH, Ding Q, Xu H, Yang B, Huangfu C, Wang Q, Liu XL, Wang XY, Wu Y, Hu X, Yuan S, Zhang Z. Microporous metal-containing hydrogen-bonded organic frameworks with benchmark C(2)H(2) storage density for efficient C(2)H(2)/C(2)H(4) and C(2)H(2)/CO(2) separations. Angew Chem Int Ed Engl 2025; 64: e202514417. 41024749 10.1002/anie.202514417

[25] Jafari H , Ghaffari-Bohlouli P, Podstawczyk D, Nie L, Shavandi A. Tannic acid post-treatment of enzymatically crosslinked chitosan-alginate hydrogels for biomedical applications. Carbohydr Polym 2022;295:119844. 35988997 10.1016/j.carbpol.2022.119844

[26] He X , Liu X, Yang J, Du H, Chai N, Sha Z, Geng M, Zhou X, He C. Tannic acid-reinforced methacrylated chitosan/methacrylated silk fibroin hydrogels with multifunctionality for accelerating wound healing. Carbohydr Polym 2020;247:116689. 32829817 10.1016/j.carbpol.2020.116689

[27] Hassani Besheli N , Verbakel J, Hosseini M, Andrée L, Joosten B, Walboomers XF, Cambi A, Yang F, Leeuwenburgh SCG. Cellular uptake of modified mesoporous bioactive glass nanoparticles for effective intracellular delivery of therapeutic agents. Int J Nanomedicine 2023;18:1599–612. 37013026 10.2147/IJN.S397297 PMC10066699

[28] Ni Y , Tian B, Lv J, Li D, Zhang M, Li Y, Jiang Y, Dong Q, Lin S, Zhao J, Huang X. 3D-printed PCL scaffolds loaded with bFGF and BMSCs enhance tendon-bone healing in rat rotator cuff tears by immunomodulation and osteogenesis promotion. ACS Biomater Sci Eng 2025;11:1123–39. 39851055 10.1021/acsbiomaterials.4c02340

[29] Ling Z , Ge X, Jin C, Song Z, Zhang H, Fu Y, Zheng K, Xu R, Jiang H. Copper doped bioactive glass promotes matrix vesicles-mediated biomineralization via osteoblast mitophagy and mitochondrial dynamics during bone regeneration. Bioact Mater 2025;46:195–212. 39760064 10.1016/j.bioactmat.2024.12.010 PMC11699476

[30] Pereyra M , Navatta M, Méndez E. Failure in the adhesion of hydroxyapatite coatings to surgical screws: a Fourier transform infrared spectroscopy qualitative study. Front Coat Dyes Interface Eng 2025;3:

[31] Jiang X , Wei J, Ding X, Zheng K, Zhou T, Shi J, Lai H, Qian S, Zhang X. From ROS scavenging to boosted osseointegration: cerium-containing mesoporous bioactive glass nanoparticles functionalized implants in diabetes. J Nanobiotechnology 2024;22:639. 39425200 10.1186/s12951-024-02865-y PMC11488221

[32] Li Q , Wang L, Li Y, Nan S, Tan Z, Yang Q, Li C, Xie X, Yan H, Hou G, Duan S, Zhao YQ. Tannic acid coating modification of polypropylene providing pH-responsive antibacterial and anti-inflammatory properties applicable to ostomy patches. Colloids Surf B Biointerfaces 2025;250:114567. 39983451 10.1016/j.colsurfb.2025.114567

[33] Jin X , Wei C, Li K, Yin P, Wu C, Zhang W. Polyphenol-mediated hyaluronic acid/tannic acid hydrogel with short gelation time and high adhesion strength for accelerating wound healing. Carbohydr Polym 2024;342:122372. 39048222 10.1016/j.carbpol.2024.122372

[34] Chen X , Liu Y, Zhao Y, Ouyang Z, Zhou H, Li L, Li L, Li F, Xie X, Hill RG, Wang S, Chen X. Halide-containing bioactive glasses enhance osteogenesis in vitro and in vivo. Biomater Adv 2022;143:213173. 36356468 10.1016/j.bioadv.2022.213173

[35] Huang C , Shi S, Qin M, Rong X, Ding Z, Fu X, Zeng W, Luo L, Wang D, Luo Z, Li Y, Zhou Z. A composite hydrogel functionalized by borosilicate bioactive glasses and VEGF for critical-size bone regeneration. Adv Sci (Weinh) 2024; 11: e2400349. 38713747 10.1002/advs.202400349 PMC11234436

[36] Kang Z , Li D, Shu C, Du J, Yu B, Qian Z, Zhong Z, Zhang X, Yu B, Huang Q, Huang J, Zhu Y, Yi C, Ding H. Polydopamine coating-mediated immobilization of BMP-2 on polyethylene terephthalate-based artificial ligaments for enhanced bioactivity. Front Bioeng Biotechnol 2021;9:749221. 34869260 10.3389/fbioe.2021.749221 PMC8636993

[37] Li YM , Wu JY, Jiang J, Dong SK, Chen YS, He HY, Liu CS, Zhao JZ. Chondroitin sulfate-polydopamine modified polyethylene terephthalate with extracellular matrix-mimetic immunoregulatory functions for osseointegration. J Mater Chem B 2020;8:8476–7. 32902534 10.1039/d0tb90152k

[38] Huang L , Gong W, Huang G, Li J, Wu J, Dong Y. The additive effects of bioactive glasses and photobiomodulation on enhancing bone regeneration. Regen Biomater 2023; 10:rbad024. 37020752 10.1093/rb/rbad024 PMC10070041

[39] Shang S , Zhuang K, Chen J, Zhang M, Jiang S, Li W. A bioactive composite hydrogel dressing that promotes healing of both acute and chronic diabetic skin wounds. Bioact Mater 2024;34:298–310. 38261910 10.1016/j.bioactmat.2023.12.026 PMC10796815

[40] Baker KC , Fleischer M, Newton MD, Galasso L, Cavinatto L, Weisz KM, Hartner S, Maerz T, Lammlin L, Baker EA, Allen AA, Bedi A. Pharmacologic mobilization and chemokine-directed recruitment of mesenchymal stromal cells to the surgically repaired rotator cuff. Am J Sports Med 2025;53:1806–16. 40444728 10.1177/03635465251341439

[41] Tan J , Liu X, Zhou M, Wang F, Ma L, Tang H, He G, Kang X, Bian X, Tang K. Effect of treadmill training on fibrocartilage complex repair in tendon-bone insertion healing in the postinflammatory stage. Bone Joint Res 2023;12:339–51. 37219405 10.1302/2046-3758.125.BJR-2022-0340.R2 PMC10204653

[42] Sohrabi M , Hesaraki S, Shahrezaee M, Shams-Khorasani A. The release behavior and in vitro osteogenesis of quercetin-loaded bioactive glass/hyaluronic acid/sodium alginate nanocomposite paste. Int J Biol Macromol 2024;280:136094. 39343279 10.1016/j.ijbiomac.2024.136094

[43] Yao H , Turali Emre ES, Fan Y, Wang J, Liu F, Wei J. L-arginine modified mesoporous bioactive glass with ROS scavenging and NO release for periodontitis treatment. Bioact Mater 2025;48:200–16. 40046013 10.1016/j.bioactmat.2025.02.015 PMC11880720

[44] Zhao H , Wang X, Jin A, Wang M, Wang Z, Huang X, Dai J, Wang X, Lin D, Shen SG. Reducing relapse and accelerating osteogenesis in rapid maxillary expansion using an injectable mesoporous bioactive glass/fibrin glue composite hydrogel. Bioact Mater 2022;18:507–25. 35415307 10.1016/j.bioactmat.2022.03.001 PMC8976096

[45] Gu J , Zhang P, Li H, Wang Y, Huang Y, Fan L, Ma X, Qian X, Xi J. Cerium-luteolin nanocomplexes in managing inflammation-related diseases by antioxidant and immunoregulation. ACS Nano 2024;18:6229–42. 38345570 10.1021/acsnano.3c09528

[46] Qian Y , Zhu J, He Y, Qin H, Qian P, Sun B, Huang H, Kuang C, Yang Q, Ou Y, Sun R, Xu F, Wang X, Luo Z, Wang Q. Bioactive siRNA-based liposomes promoted tendon-bone healing in osteoporotic mice by recovering the stemness of CD248(+) TSPCs. Adv Sci (Weinh) 2025; 12: e09883. 40536433 10.1002/advs.202509883 PMC12442593

[47] Zhu D , Zhu X, Zhang Y, Huang X. Leptin receptor signaling mediates the distinct tendon-bone interface reconstruction in rotator cuff tears and osteoporosis-comorbid rotator cuff tears. Stem Cell Res Ther 2025;16:449. 40847364 10.1186/s13287-025-04586-x PMC12374385

[48] Dang W , Wang X, Li J, Deng C, Liu Y, Yao Q, Wang L, Chang J, Wu C. 3D printing of Mo-containing scaffolds with activated anabolic responses and bi-lineage bioactivities. Theranostics 2018;8:4372–92. 30214627 10.7150/thno.27088 PMC6134938

[49] Han J , Han SC, Jeong HJ, Rhee SM, Kim YS, Jin YJ, Park SH, Oh JH. Recombinant human parathyroid hormone biocomposite promotes bone-to-tendon interface healing by enhancing tenogenesis, chondrogenesis, and osteogenesis in a rabbit model of chronic rotator cuff tears. Arthroscopy 2024;40:1093–104.e2. 38000485 10.1016/j.arthro.2023.09.034

[50] Sun J , Chen QZ, Hong AZ, Ju F, Wang HL, Zhang B, Liu W, Sun YC, Tan J, Yang QQ, Zhou YL. Dissolvable microneedle-assisted transdermal administration of diacerein nanoparticles achieved satisfactory therapeutic effects in tendon-bone insertion repair by reducing oxidative stress and inflammation. Mater Today Bio 2025;33:101999. 10.1016/j.mtbio.2025.101999 PMC12240096 40636026

[51] Liu S , Liu W, Yang Q, Yang S, Yang Y, Fan L, Zhang Y, Qi B, Shi Z, Wei X, Zhu L, Li T. Non-coding-RNA-activated core/chitosan shell nanounits coated with polyetheretherketone for promoting bone regeneration and osseointegration via osteoimmunology. ACS Appl Mater Interfaces 2023;15:12653–68. 36868875 10.1021/acsami.2c19186

[52] Zheng X , Luo H, Li J, Yang Z, Zhuan X, Li X, Chen Y, Huo S, Zhou X. Zinc-doped bioactive glass-functionalized polyetheretherketone to enhance the biological response in bone regeneration. J Biomed Mater Res A 2024;112:1565–77. 38514993 10.1002/jbm.a.37710

[53] Jia N , Gao Y, Li M, Liang Y, Li Y, Lin Y, Huang S, Lin Q, Sun X, He Q, Yao Y, Zhang B, Zhang Z, Zhang L. Metabolic reprogramming of proinflammatory macrophages by target delivered roburic acid effectively ameliorates rheumatoid arthritis symptoms. Signal Transduct Target Ther 2023;8:280. 37500654 10.1038/s41392-023-01499-0 PMC10374631

[54] Qin B , Bao D, Liu Y, Zeng S, Deng K, Liu H, Fu S. Engineered exosomes: a promising strategy for tendon-bone healing. J Adv Res 2024;64:155–69. 37972886 10.1016/j.jare.2023.11.011 PMC11464473

