# Supplementary material for: ROS scavenging nanoengineered bioactive glass interfaces reprogram macrophage immunity for tendon–bone regeneration
Source: Regen Biomater. 2026 Jun 9;13:rbag121. doi: 10.1093/rb/rbag121 (PMC13332430; doi:10.1093/rb/rbag121)
Supplement: rbag121_Supplementary_Data [file rbag121_supplementary_data.docx]

**Supplementary data**

**ROS-Scavenging Nanoengineered Bioactive Glass Interfaces Reprogram Macrophage Immunity for Tendon-Bone Regeneration**

Bowen Cai^1,2,#^, Fanrui Zeng^3,4,#^, Kaixiao Xue^1,2,#^, Zhi Shen^1,2^, Chang Qiao^1,2^, Han Wu^3,4^, Qunyi Wang^5^, Jue Zhang^6^, Shahin Homaeigohar^7^, Kai Zheng^3,4,*^, Bin Zhu^1,2,*^, Jiahu Fang^1,2,*^

^1^Department of Orthopedics, First Affiliated Hospital with Nanjing Medical University, Nanjing 210029, China

^2^Jiangsu Institute of Functional Reconstruction and Rehabilitation, Nanjing 210029, China

^3^Jiangsu Province Engineering Research Center of Stomatological Translational Medicine& Jiangsu Key Laboratory of Oral Diseases, Nanjing Medical University, Nanjing 210029, China

^4^Affiliated Hospital of Stomatology, Nanjing Medical University, Nanjing 210029, China

^5^Nanjing Stomatological Hospital, Affiliated Hospital of Medical School, ResearchInstitute of Stomatology, Nanjing University, Nanjing 210029, China

^6^Anhui Province Engineering Research Center for Dental Materials and Application, School of Stomatology, Wannan Medical College, Wuhu 241002, China

^7^School of Science and Engineering, University of Dundee, DD1 4HN, United Kingdom.

^#^The first three authors contributed equally to this work.

*^*^*Correspondence address. E-mail: kaizheng@njmu.edu.cn (K.Z.); [zhubin@njmu.edu.cn (B.Z.);](mailto:zhubin@njmu.edu.cn%20(B.Z.);) [fjh4508@163.com](mailto:fjh4508@163.com) (J.F.)


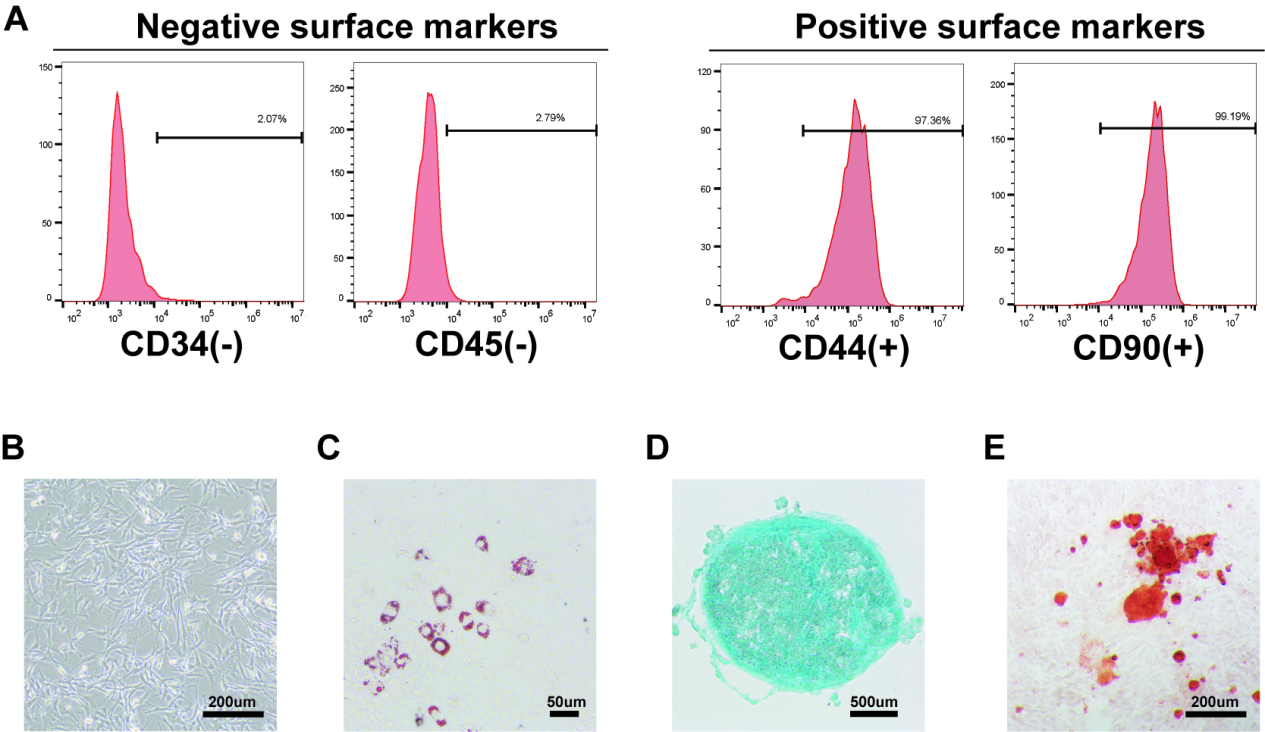


**Figure S1**. **Identification of rat bone marrow mesenchymal stem cells.** A) CD34, CD45, CD44, CD90 of mesenchymal stem cells were detected by flow cytometry. B）Microscopic images of BMSCs. C-E）Pictures of trilineage differentiation of BMSCs (oil O staining, toluidine blue staining, and alizarin red staining).

**Table S1.** Primer sequences used in qPCR.

| **Primers Sequence** | |
| --- | --- |
| COL1A1  OCN  RUNX2  COL2A1  ACAN  SOX9  TNF-α  IL-1β  CCL-2  GAPDH | Forward: CCTTCTGGTCCTCGTGGTCTCC  Reverse: AGCCTCGGTGTCCCTTCATTCC  Forward: CCTCACTCTTGTCGCCCTG  Reverse: CTCTTGGACACGAAGGCTGA  Forward: CCTTCCACTCTCAGTAAGAAGA  Reverse: TAAGTAAAGGTGGCTGGATAGT  Forward: CACGCTCAAGTCGCTGAACAAC  Reverse: CACGCTCAAGTCGCTGAACAAC  Forward: AGTGCTATGCTGGCTGGTTGG  Reverse: ATGGTGCTTGGACAGTGGATCAG  Forward: GAGGCCACGGAGCAGACGCA  Reverse: CAGCGCCTTGAAGATGGCGTT  Forward: ACGTGGAACTGGCAGAAGAGG  Reverse: TGAGAAGAGGCTGAGACATAGGC  Forward: ACGTGGAACTGGCAGAAGAGG  Reverse: TGAGAAGAGGCTGAGACATAGGC  Forward: CACTCACCTGCTGCTACTCATTC  Reverse: CACTCACCTGCTGCTACTCATTC  Forward: CCGCATCTTCTTGTGCAGTG  Reverse: CGATACGGCCAAATCCGTTC |


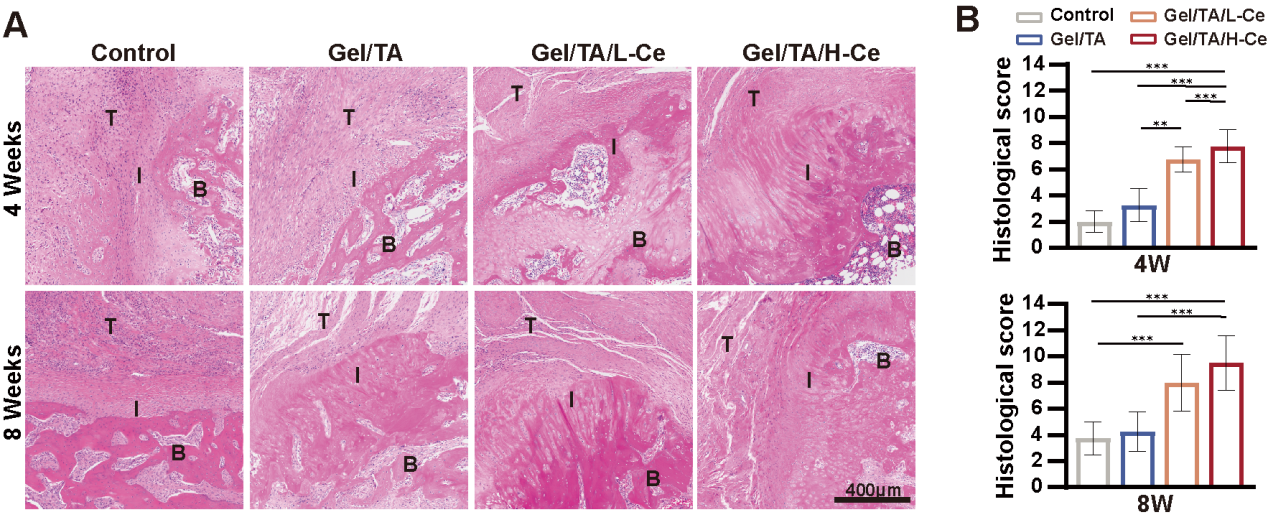


**Figure S2. HE staining of the tendon-bone interface**. A) HE staining of the tendon-bone interface. B) Histological scores of the control, Gel/TA, Gel/TA/L-Ce, and Gel/TA/H-Ce groups. T: tendon, I: tendon-bone interface, B: bone. (*p < 0.05, **p < 0.01, and ***p < 0.001).
